# Imaging of a van der Waals spin-orbit torque system using spin ensembles in hBN

**DOI:** 10.1038/s41467-026-74178-7

**Published:** 2026-06-10

**Authors:** Xi Zhang, Jingcheng Zhou, Chaowei Hu, PeiYu Cai, Kuangyin Deng, Chuangtang Wang, Nishkarsh Agarwal, Hanshang Jin, Faris A. Al-Matouq, Stelo Xu, Roshan S. Trivedi, Senlei Li, Sumedh Rathi, Hanyi Lu, Zhigang Jiang, Valentin Taufour, Robert Hovden, Liuyan Zhao, Ran Cheng, Xiaodong Xu, Elton J. G. Santos, Jiun-Haw Chu, Chunhui Rita Du, Hailong Wang

**Affiliations:** 1 School of Physics, Georgia Institute of Technology, Atlanta, GA USA; 2 Department of Physics, University of Washington, Seattle, WA USA; 3 Institute for Condensed Matter and Complex Systems, School of Physics and Astronomy, University of Edinburgh, Edinburgh, UK; 4 Department of Electrical and Computer Engineering, University of California, Riverside, CA USA; 5 Department of Physics, University of Michigan, Ann Arbor, MI USA; 6 Department of Materials Science and Engineering, University of Michigan, Ann Arbor, MI USA; 7 Department of Physics and Astronomy, University of California, Davis, CA USA; 8 Department of Physics, University of California, San Diego, La Jolla, CA USA; 9 Applied Physics Program, University of Michigan, Ann Arbor, MI USA; 10 Department of Physics and Astronomy, University of California, Riverside, CA USA; 11 Department of Materials Science and Engineering, University of Washington, Seattle, WA USA; 12 Higgs Centre for Theoretical Physics, University of Edinburgh, Edinburgh, UK; 13 Donostia International Physics Center, Donostia-San Sebastián, Spain

**Keywords:** Two-dimensional materials, Spintronics, Quantum metrology, Magnetic devices, Imaging techniques

## Abstract

Recently, optically active spin defects embedded in two-dimensional (2D) van der Waals (vdW) crystals have emerged as a transformative quantum sensing platform to explore cutting-edge materials science. Taking advantage of excellent solid-state integrability, this new class of spin defects can be readily arranged in nanoscale proximity to target materials, showing great promise for realizing in-situ quantum sensing of microscopic spin and charge behaviors in vdW heterostructures. Here we report hexagonal boron nitride-based quantum imaging of field-free deterministic magnetic switching and electric current distributions in an all-vdW spin-orbit torque (SOT) system. By visualizing variations of nanoscale magnetic stray field profile of room-temperature 2D magnet Fe_3_GaTe_2_ under different SOT conditions, we show how the magnetic switching evolves from deterministic to stochastic behavior due to the interplay between spin orientations, anisotropy and Joule heating. Micromagnetic simulations rationalize our results well, revealing the role of field-like SOT in inhibiting thermal fluctuation driven stochastic switching and chaotic multi-domain competition. This understanding, which is otherwise difficult to access by conventional transport measurements, offers valuable insights into material design, testing, and performance evaluation of next-generation vdW spintronic devices.

## Abstract (web summary)

Recently field-free spin-orbit torque switching has been demonstrated in van der Waals magnetic materials. Here, taking advantage of the quantum sensing properties of spin defects in hexagonal Boron Nitride, Zhang, Zhou, and coauthors image the current-induced spin-orbit torque switching in the van der Waals magnet, Fe3GaTe2

## Introduction

Spin-orbit torques (SOTs) have been widely used for developing fast, scalable, and energy-efficient magnetization control in modern spintronic memory devices^1,2^. The flourishing van der Waals (vdW) crystals provide an appealing material playground to revolutionize the conventional SOT technologies^3–6^. Potential advantages associated with the emerging two-dimensional (2D) spintronic devices include highly engineerable vdW interfacial conditions, tunable polarization of injected spin currents, convenient material co-integrations, and readily established magnetic vdW proximity, opening new pathways for designing ultracompact spin logic circuits with tailored functionalities and hybrid performances^3,7^. The recently observed out-of-plane spin-driven field-free deterministic magnetic switching of a perpendicular vdW magnet is a notable example in this catalog^8–11^. The elimination of auxiliary magnetic fields is particularly favorable for implementing scalable spin memory device applications with reduced energy consumption^8,10–13^.

Despite the remarkable progress made thus far, ongoing research on SOT-driven field-free magnetic switching in vdW heterostructures has mainly focused on magneto-transport measurements. These studies are susceptible to thermal, electromigration, and magnetoelastic artifacts^8–11^, which can in principle be resolved by nanoscale imaging of magnetization switching details. However, implementation of such cutting-edge imaging techniques in all-vdW SOT systems remains an open challenge in the current state of the art. This limitation hinders a comprehensive understanding of the fundamental magnetic switching mechanism on 2D flatland and impedes future improvement of device performance.

Here, we report a multimodal vdW device platform endowed with dual quantum sensing and electrical SOT functionalities to address these technical challenges. Taking advantage of optically active spin defects in hexagonal boron nitride (hBN), we demonstrate self-integrated quantum imaging^14–17^ of field-free deterministic magnetization switching in WTe_2_/Fe_3_GaTe_2_/hBN vdW heterostructures. Using spin ensembles embedded in the hBN encapsulation layer^17–21^, we directly visualize the microscopic Fe_3_GaTe_2_ stray field profile as a function of electrical switching currents, revealing systematic evolutions of magnetic domains at different SOT conditions. Spatial imaging of microscopic electric current flow is also realized in the presented multifunctional vdW device platform, and it shows a close correlation with the observed field-free magnetic switching behavior. We further perform micromagnetic simulations to reveal that a robust field-like SOT could provide a low-energy path for local domain wall nucleation and propagation in Fe_3_GaTe_2_, inhibiting thermal fluctuation-driven stochastic switching and chaotic multi-domain competition. Our study enriches the current understanding of SOT-induced magnetization dynamics in vdW heterostructures, bringing new opportunities for investigating the microscopic electromagnetic properties of 2D spintronic devices^22^.

## Results and discussions

### Self-integrated vdW quantum sensing platform

**Fig. 1:**
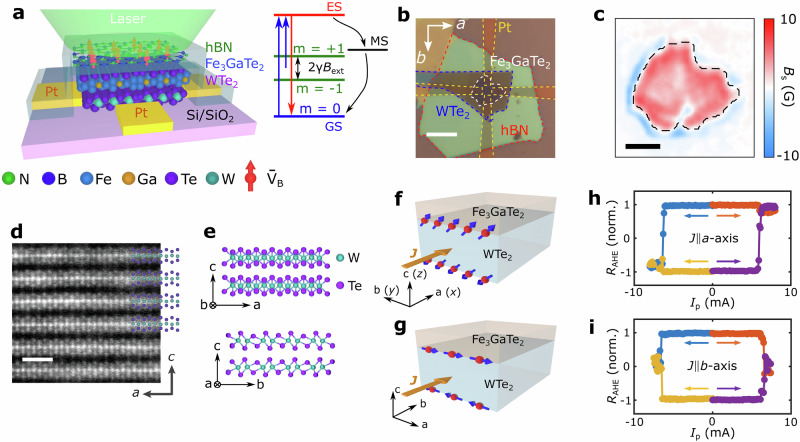
A multimodal vdW device platform for dual quantum imaging and electrical SOT measurements. **a** Left: Schematic of the WTe_2_/Fe_3_GaTe_2_/hBN vdW heterostructure for quantum sensing and electrical SOT switching measurements. Right: Energy level diagram of a ${V}_{{{\rm{B}}}}^{-}$ spin defect and optical excitation (blue arrow), radiative recombination (red arrow), and nonradiative decay (black arrow) between the ground state (GS), excited state (ES), and metastable state (MS). **b** Optical microscopy image of a prepared WTe_2_/Fe_3_GaTe_2_/hBN device. Boundaries of vdW layers are outlined by red (hBN), blue (WTe_2_), and white (Fe_3_GaTe_2_) dashed lines. Pt electrodes are highlighted by yellow dashed lines. The scale bar is 10 μm. **c** hBN wide-field imaging of out-of-plane magnetic stray field *B*_s_ generated by the Fe_3_GaTe_2_ sample. The measurement temperature is 260 K with an out-of-plane oriented external magnetic field *B*_ext_ of 30 G applied. Black dashed lines outline the boundary of the Fe_3_GaTe_2_ flake. The scale bar is 4 μm. **d** Cross-sectional HAADF-STEM image of a separately prepared device viewed along the *b*-axis of WTe_2_. The highlighted atomic structure shows the broken lateral mirror symmetry in the *ac*-plane of WTe_2_. The scale bar is 1 nm. **e** Atomic arrangement of WTe_2_ viewed along its *b*-axis (top) and *a*-axis (bottom) directions. **f**,**g** Generation of out-of-plane and in-plane polarized spin currents when an electrical charge current *J* flows along the low-symmetry and high-symmetry crystallographic axes of WTe_2_. **h**,**i** Normalized anomalous Hall resistance of Fe_3_GaTe_2_ measured as a function of electrical write current pulse *I*_p_ applied *a*long the *a*-axis (**h**) and *b*-axis (**i**) of WTe_2_. The magnitude of *I*_p_ is increased from zero with the sweeping direction indicated by the color arrows. Fe_3_GaTe_2_ is initialized to the *m*_z_ = 1 or *m*_z_ = $-$1 state in the beginning of each sweeping sequence. External auxiliary magnetic fields are absent in presented SOT measurements.

We first review the vdW heterostructures used in the current study for hBN quantum sensing and electrical SOT measurements as illustrated in Fig. 1a. Exfoliated WTe_2_, Fe_3_GaTe_2,_ and hBN nanoflakes with desirable thicknesses and lateral dimensions are picked up in sequence to form WTe_2_/Fe_3_GaTe_2_/hBN stacking devices and then are released onto Si/SiO_2_ substrates with patterned platinum (Pt) electrodes. The thicknesses of WTe_2_ and Fe_3_GaTe_2_ layers are ~10 nm and ~7 nm to balance the interfacial spin accumulation and electrical shunting effect (see Methods Section and Supplementary Information Note 1 for details). Figure 1b presents the optical microscopy image of a prepared WTe_2_/Fe_3_GaTe_2_/hBN vdW device (device A). It is worth mentioning that Fe_3_GaTe_2_ is a recently discovered vdW magnet with a spontaneous perpendicular magnetic anisotropy and bulk Curie temperature exceeding 350 K^23–25^. Few-layer-thick Fe_3_GaTe_2_ shows robust magnetic order above room temperature (see Supplementary Information Note 1 for details), serving as an excellent material candidate to develop functional vdW spintronic logic devices^10,23,24^.

The hBN encapsulation layer with a thickness of tens of nanometers is utilized to prevent sample degradation and simultaneously serves as an in-situ 2D quantum sensing platform. It contains boron vacancy spin defects ${V}_{{{\rm{B}}}}^{-}$ created by thermal neutron irradiation method (see Methods Section for details)^26^. Each boron atom vacancy ${V}_{{{\rm{B}}}}^{-}$ is surrounded by three nearest-neighboring nitrogen atoms, and its energy level structure is shown in the right panel of Fig. 1a. The negatively charged ${V}_{{{\rm{B}}}}^{-}$ defect possesses an *S* = 1 ground state electron spin with an intrinsic quantization axis along the out-of-plane direction. The Zeeman effect-induced “three-level” quantum system can be optically addressed by measuring the spin-dependent ${V}_{{{\rm{B}}}}^{-}$ photoluminescence (PL)^17,18,27,28^. The magnitude of local static magnetic field parallel to the ${V}_{{{\rm{B}}}}^{-}$ spin axis can be deduced from the energy splitting between PL peaks corresponding to the m_s_ = $+$1 and m_s_ = $-$1 spin state(s)^14–16^ (see Supplementary Information Note 3 for details). To achieve nanoscale quantum imaging of Fe_3_GaTe_2_ nanoflakes, we utilize wide-field quantum magnetometry to detect fluorescence of ${V}_{{{\rm{B}}}}^{-}$ ensembles across the field of view projected on a CMOS camera^14,15^. Figure 1c shows an out-of-plane magnetic stray field *B*_s_ map of the prepared vdW stacking device (device A). One can see that an atomically thin (~6.3 nm) Fe_3_GaTe_2_ sample shows robust magnetization at 260 K, in agreement with the magneto-transport studies^10,24^. The demonstrated hBN quantum imaging platform provides an attractive viewport to investigate the microscopic spin properties of 2D vdW SOT devices.

### Electrical detection of unconventional SOTs

In the prepared all-vdW heterostructure, WTe_2_ serves as the spin source material whose broken crystal symmetry allows generation of spin currents with tunable spin orientations^8–10,29^. Figure 1d shows a cross-sectional high-angle annular dark-field scanning transmission electron microscopy (HAADF-STEM) image of the WTe_2_ layer viewed along its *b*-axis. WTe_2_ has a mirror symmetry with respect to the *bc*-plane but not the *ac*-plane, as illustrated in Fig. 1e. When an electrical charge current is applied along the low-symmetry crystallographic axis (*a*-axis) of WTe_2_, the broken symmetry enables generation of spin currents with out-of-plane polarization in addition to the conventional spin Hall effect (Fig. 1f)^8–10,29^. When an in-plane charge current flows along the high-symmetry axis (*b*-axis), where the mirror symmetry with respect to the *bc*-plane is preserved, the spin Hall effect dominates in this situation, and the directions of the electric field, spin currents, and spin polarization are mutually orthogonal, as shown in Fig. 1g^8,29^. In the current work, the crystal symmetry and orientation of WTe_2_ are evaluated by rotational anisotropy second harmonic generation (RA-SHG) measurements^30–34^ (see Supplementary Information Note 4 for details).

We now present electrical SOT measurement results on field-free deterministic magnetic switching of perpendicular Fe_3_GaTe_2_ magnetization. Figure 1h shows the current-driven anomalous Hall loop of the Fe_3_GaTe_2_ sample without the assistance of an external magnetic field (see Methods Section and Supplementary Information Note 5 for details). The electric current is applied along the low-symmetry axis of WTe_2_, and the measurement temperature is 200 K. We observe robust field-free deterministic magnetic switching, where the measured Hall voltage signals of Fe_3_GaTe_2_ show positive/negative jumps at the critical write current pulses, and the terminal magnetic state is solely determined by the current polarity. Field-free bipolar switching of the perpendicular vdW magnet is attributed to out-of-plane spins injected from WTe_2_, which effectively compensate for the local magnetic damping and overcome the anisotropy of the Fe_3_GaTe_2_ magnetization. By measuring the shift of anomalous Hall loops to characterize the equivalent out-of-plane magnetic field produced by spin currents from WTe_2_, the unconventional SOT efficiency is estimated to be 0.180 $\pm$ 0.045 in the all-vdW material system presented (see Supplementary Information Note 6 for details).

In contrast, the signature of field-free deterministic magnetic switching vanishes when electric current pulses are applied along the high-symmetry crystallographic axis (*b*-axis) of WTe_2_, as shown in Fig. 1i. Without the assistance of out-of-plane spins or an auxiliary in-plane magnetic field breaking the time reversal symmetry, the Fe_3_GaTe_2_ magnetization is driven to an intermediate phase that is in-plane magnetized or thermally randomized under large positive and negative current pulses (see Supplementary Information Note 5 for details). The in-plane antidamping SOT could tilt the Fe_3_GaTe_2_ magnetization to the sample plane^8,35^ followed by spontaneous out-of-plane self-remagnetization after electrical write current pulses are turned off, leading to fully randomly oriented magnetic domains without showing a total net perpendicular magnetization^8^. Current-induced Joule heating effect may also be involved in this process by driving thermal demagnetization in the Fe_3_GaTe_2_ sample; nevertheless, the overall “two-step” out-of-plane demagnetization-remagnetization physical picture remains valid due to the lack of symmetry-breaking fields to realize deterministic perpendicular switching^8,35^.

### Visualizing field-free deterministic magnetic switching in the all-vdW SOT system

**Fig. 2:**
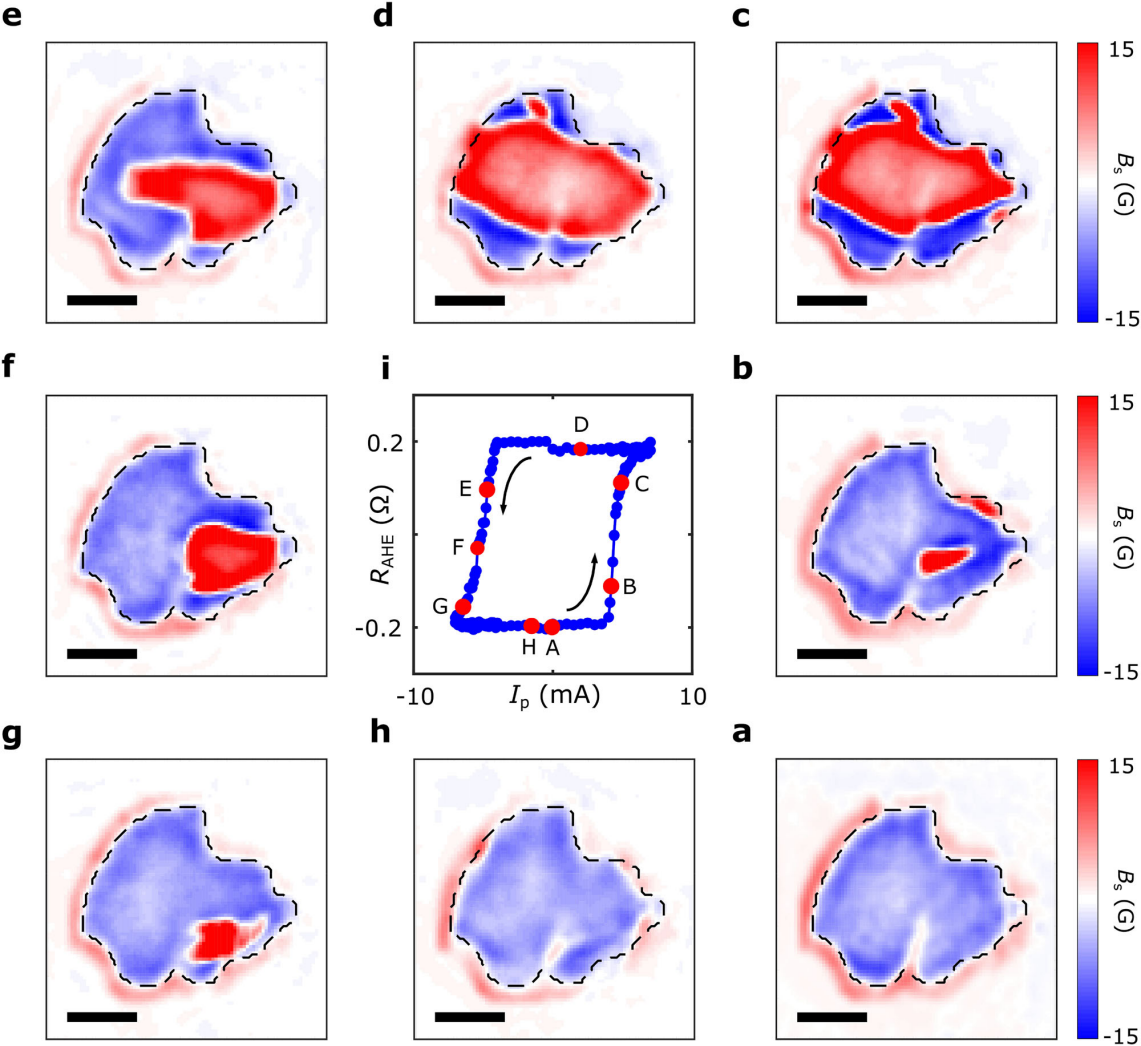
Visualizing field-free deterministic magnetic switching of Fe_3_GaTe_2_. **a–h** hBN-based quantum imaging of microscopic evolutions of Fe_3_GaTe_2_ magnetic domains during the field-free deterministic magnetic switching process. The scale bar is 4 μm. **i**, Anomalous Hall resistance of Fe_3_GaTe_2_ measured as a function of electrical write current pulse *I*_p_ in absence of an external magnetic field. The arrows indicate that *I*_p_ is swept from zero following the counterclockwise direction around the hysteresis loop and finally returns to the starting point. hBN-based quantum imaging measurements presented in Figs. 2a–h are performed at the corresponding points from “A” to “H” on the field-free deterministic switching loop (Fig. 2i). The SOT-driven magnetic switching and quantum sensing measurements are performed at 260 K.

We now utilize hBN-based wide-field quantum microscopy^14,15^ to visualize the field-free deterministic magnetic switching of Fe_3_GaTe_2_ at the nanoscale. Figures 2a–h present a series of representative stray field maps of the Fe_3_GaTe_2_ sample measured at the corresponding points (“A” to “H”) on the unconventional SOT-driven magnetic hysteresis loop. Electric current pulses *I*_p_ are applied along the low-symmetry axis of WTe_2_ to ensure the field-free nature of the observed deterministic magnetic switching (Fig. 2i). One can see that the Fe_3_GaTe_2_ sample starts from one of the spontaneous magnetic easy states with magnetization pointing down, producing a negative stray field at point “A” (see Supplementary Information Note 7 for details). When the electrical write current pulse *I*_p_ is below the threshold value, the measured stray field *B*_s_ map basically remains the same, indicating a negligible effect of out-of-plane spins on the Fe_3_GaTe_2_ magnetization. When ramping *I*_p_ above the critical value, out-of-plane antidamping SOT starts to locally flip the perpendicular Fe_3_GaTe_2_ magnetization, generating a stray field with opposite polarity. The observed field-free magnetic switching starts from magnetic domains at locations where the energy barrier is the lowest and then extends to neighboring Fe_3_GaTe_2_ sample areas, as shown in Fig. 2b–d. Here, we note that Joule heating and/or defects induced local variations of the Fe_3_GaTe_2_ magnetization could assist in the initial domain wall nucleation processes, which typically happen in the high current-density sample areas defined by Pt electrodes^1^. When reaching the top plateau value of the anomalous Hall loop, ~70% portion of the Fe_3_GaTe_2_ magnetic domain is flipped (Fig. 2d) in the absence of an external magnetic field, consistent with our magneto-transport measurement results. It is worth mentioning that some edge areas of the Fe_3_GaTe_2_ flake are not magnetically switchable, which is attributed to the combined effect of sample size, local electric current flow, magnetic anisotropy, pinning effect, and others. Inverting electrical write current pulses into the negative regime leads to a reversal of the magnetic switching polarity accompanied by retraction of the flipped Fe_3_GaTe_2_ domains (Figs. 2e-2g). Sweeping *I*_p_ back to the starting point (“A”) completes the entire anomalous Hall loop, and the measured Fe_3_GaTe_2_ stray field map is almost identical to that measured in the initial magnetic state. The presented hBN wide-field quantum imaging measurements directly visualize the out-of-plane spin-driven field-free deterministic magnetic switching of an atomically thin vdW magnet, revealing SOT-induced nanoscale domain wall nucleation and propagation on 2D flatland. Field-free deterministic magnetic switching is also observed and imaged in other WTe_2_/Fe_3_GaTe_2_/hBN devices to confirm the consistency of the results presented (see Supplementary Information Notes 8 and 9 for details).

Here, we briefly comment on the relationship between SOT switching efficiency and flake size from a theoretical perspective. In the macroscopic scale relevant to our experiments, where device dimensions are significantly larger than the exchange length and domain wall width, the critical current density is governed primarily by the energy barrier to nucleate a reversed domain. As the sample edges are spatially decoupled from the bulk in terms of short-range exchange interactions, this nucleation threshold is determined by local edge properties such as demagnetization gradients and defects, rather than the total surface area of the sample. Therefore, for macroscopic flakes, the switching efficiency is expected to remain relatively independent of lateral size. However, if the device dimensions are reduced to the nanometer scale (e.g., sub-100 nm), finite size effects would become much more significant due to long-range dipolar interactions extending across the entire device, modifying the effective anisotropy and making the switching efficiency size dependent.

### Role of damping-like torque vs field-like torque in field-free deterministic magnetic switching

**Fig. 3:**
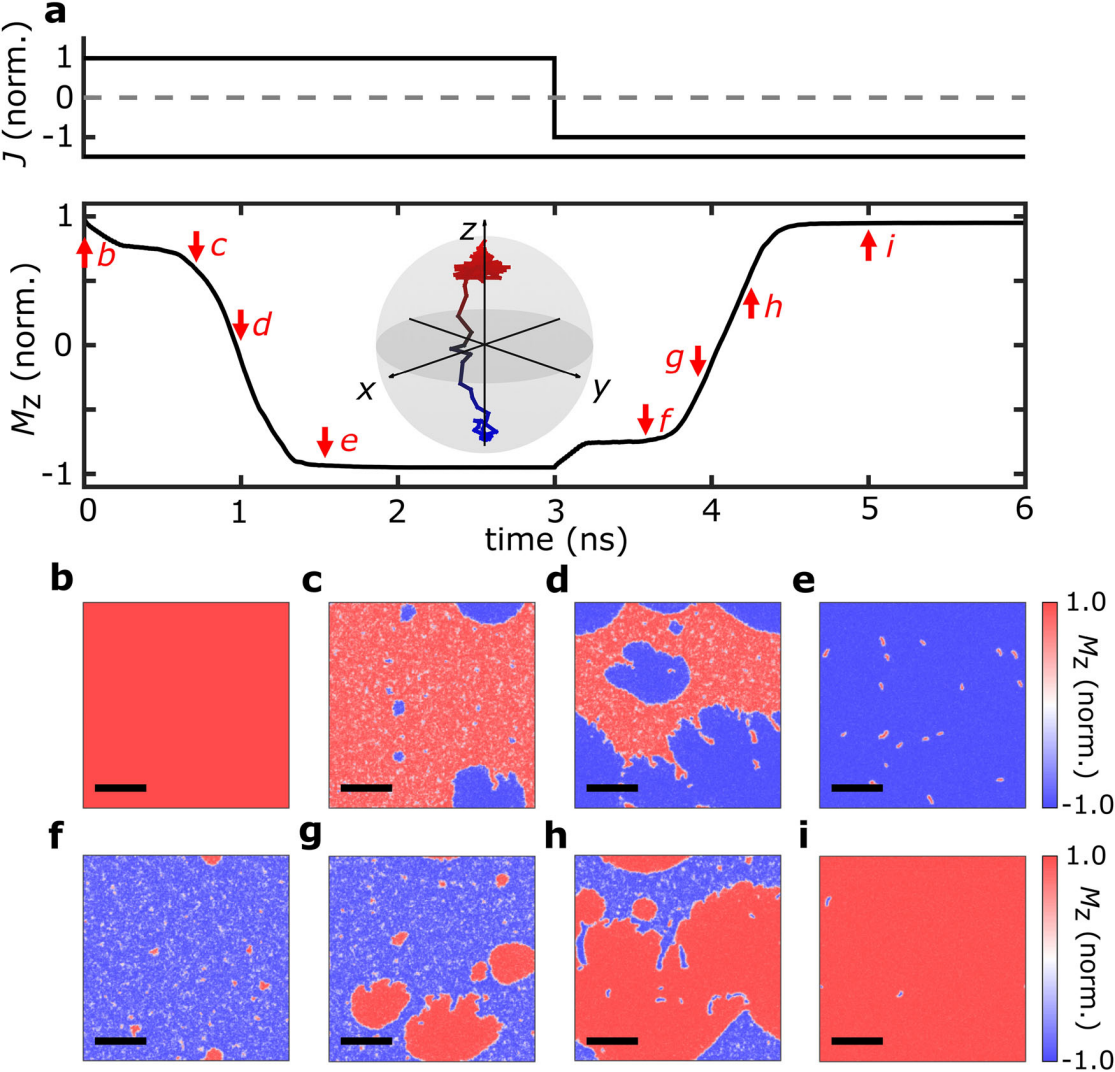
Micromagnetic simulations. **a** Modeling of the field-free perpendicular magnetic switching of Fe_3_GaTe_2_ driven by electric current pulses with an amplitude of *J* = 1.7 $\times$ 10^11 ^A/m^2^ flowing along the *a*-axis of WTe_2_. Normalized current *J* (top panel) and magnetization *M*_z_ (bottom panel) are shown along the evolution of the spins over time. The inset in the bottom panel displays the 3D path of magnetization reversal trajectories smoothed by averaging a 9 $\times$ 9 block of Fe_3_GaTe_2_ magnetic moments at the sample’s center to minimize stochastic effects from thermal fluctuations. **b–i** Screenshots at different times of the simulated spatially resolved local domain wall nucleation and propagation during the forward (**b-e**) and backward (**f–i**) field-free magnetization switching process of Fe_3_GaTe_2_. Images presented in (**b–i**) follow the dynamics shown in **a** (bottom panel). Field-like torque efficiency $\eta$_FL_ characterized by the ratio between the field-like SOT and damping-like SOT is set to be 0.5 in the presented simulations, and other simulation parameters are available in the Method Section. Note that variations of $\eta$_FL_ between 0.2 and 0.5 provide minor modifications to the simulation results. The scale bar is 0.5 µm.

After showing the electrical transport and wide-field quantum imaging results, we next perform micromagnetic simulations to further understand the underlying mechanism of the observed unconventional SOT-driven field-free Fe_3_GaTe_2_ magnetization switching. Here, we focus on the situation where electrical write current pulses are applied along the low-symmetry crystallographic axis (*a*-axis) of WTe_2_. Note that the *a-*, *b-*, and *c-*crystallographic axes of WTe_2_ correspond to *x-*, *y-*, and *z*-axes in the local coordinate frame, as shown in Fig. 1f. The effective spin polarization ***σ*** considering the combined effects of reduced crystal symmetry and spin-orbit interaction, lies in the *y-z* plane with a canting angle of 45^o^ relative to the *y*-axis in our simulations. Figure 3a presents simulated Fe_3_GaTe_2_ magnetization switching trajectories in the time domain with an electric current density *J* = $\pm$ 1.7 $\times$ 10^11 ^A/m^2^ in WTe_2_ and the field-like torque efficiency $\eta$_FL_ set to be 0.5. The out-of-plane magnetization *M*_z_ of Fe_3_GaTe_2_ shows the deterministic forward and backward switching signatures under positive and negative electrical write current application. During the initial ~0.5 ns of the current pulse duration, SOT perturbs the perpendicular Fe_3_GaTe_2_ magnetization off its equilibrium state and initiates a precessional incubation period. Then, the damping-like SOT [${{\boldsymbol{m}}}\times \left({{\boldsymbol{m}}}\times {{\boldsymbol{\sigma }}}\right)$] drives the system to gain enough energy and overcome the energy barrier imposed by the uniaxial anisotropy of Fe_3_GaTe_2_. Here, ***m*** represents the magnetic moment of Fe_3_GaTe_2_. Once the barrier is surpassed, a rapid and complete perpendicular magnetization reversal is triggered within one nanosecond, and the Fe_3_GaTe_2_ moment remains stable in the switched state afterwards. Figure 3b–e present the visual inspection of simulated forward magnetic switching of Fe_3_GaTe_2_. One can see that the magnetization reversal initiates with the nucleation of small local domain drops. The flipped magnetic patches then rapidly expand and merge via domain wall propagation (see Supplementary Video 1). When the polarity of the applied electric current is inverted, and thus the out-of-plane damping-like SOT has an opposite direction, a backward switching event is activated as shown in Fig. 3f–i.

While the damping-like SOT serves as a driving force for the observed field-free magnetic switching, it is instructive to highlight that the field-like SOT component $\left({{\boldsymbol{m}}}\times {{\boldsymbol{\sigma }}}\right)$ is helpful for establishing stable precessional magnetization dynamics of Fe_3_GaTe_2_ to ensure the deterministic nature of the switching process. A strong field-like SOT $\left({{\boldsymbol{m}}}\times {{\boldsymbol{\sigma }}}\right)$ exploits intrinsically weaker demagnetization fields at the real sample edges to shape the energy landscape of the Fe_3_GaTe_2_ flake, providing a deterministic, low-energy path to assist in incipient domain wall nucleation and follow-up propagation swiftly and coherently across the sample. In its absence, magnetic switching evolves into a stochastic process, which relies on random thermal fluctuations to overcome the more uniform energy barrier, leading to chaotic multi-domain competition. Our simulations reveal that the magnetic switching dynamics of Fe_3_GaTe_2_ become far more chaotic when the field-like SOT contribution ($\eta$_FL_) is vanishingly small in comparison with its damping-like counterpart. In contrast to the steady domain wall nucleation and propagation shown in Fig. 3, the switching proceeds via the stochastic nucleation of many small, competing domains throughout the Fe_3_GaTe_2_ sample. These domains struggle against each other, leading to a noisy and fluctuating spatially averaged magnetization that fails to reach a fully switched state during the electric current pulse application (see Supplementary Information Note 10 for details). Our micromagnetic simulations qualitatively recapture the field-free magnetization switching observed in WTe_2_/Fe_3_GaTe_2_/hBN vdW heterostructure, shedding light on the role of field-like SOT in helping achieve the desirable deterministic nature.

### From deterministic to probabilistic field-free magnetic switching

**Fig. 4:**
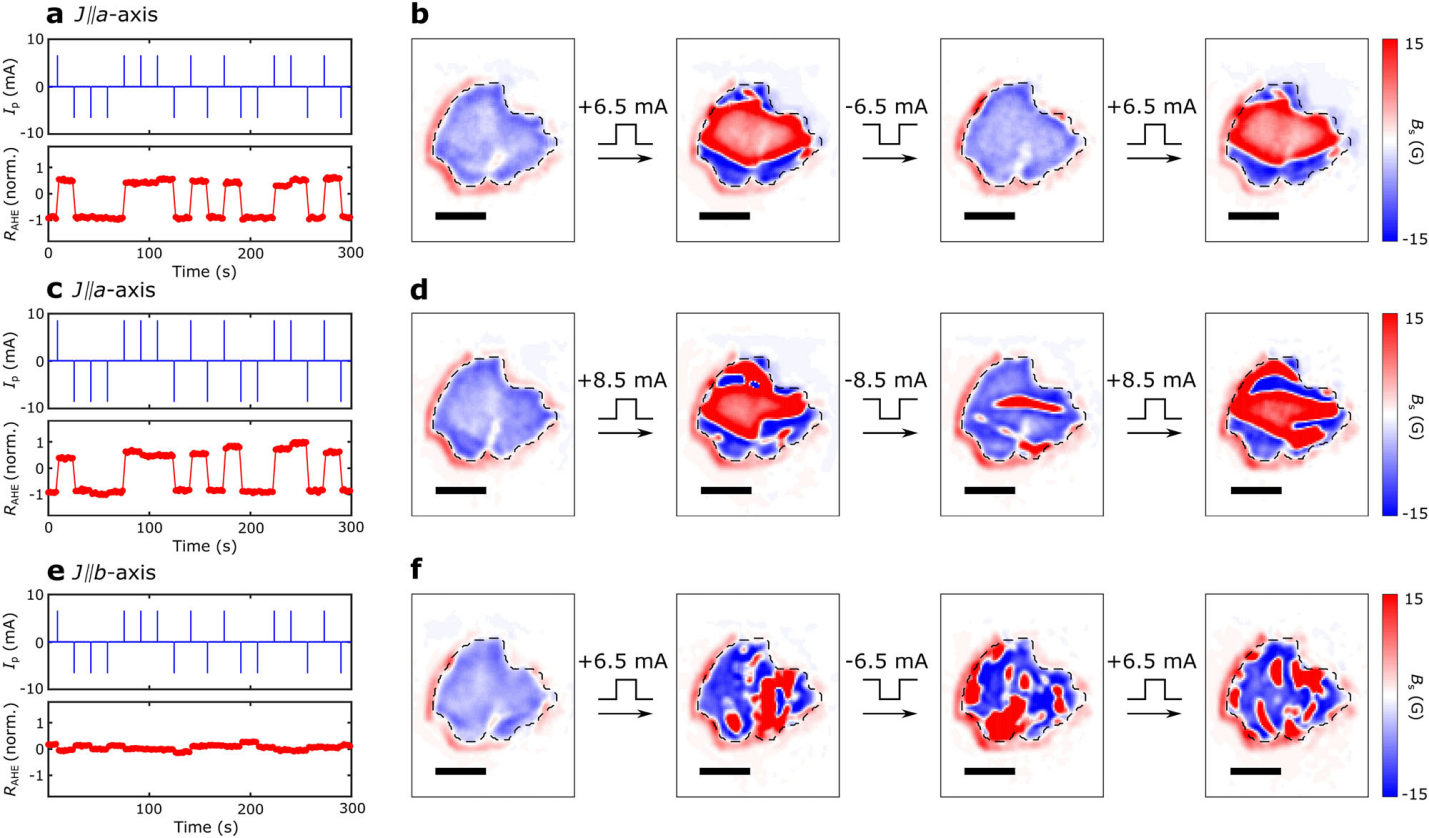
Evaluating crystal symmetry dependent field-free SOT control of Fe_3_GaTe_2_ magnetization. **a**,**c** Field-free deterministic magnetic switching of Fe_3_GaTe_2_ driven by a train of positive and negative electric current pulses (*I*_p_) with a magnitude of 6.5 mA (**a**) and 8.5 mA (**c**) along the *a*-axis of WTe_2_. **b**,**d** Variations of magnetic stray field patterns of the Fe_3_GaTe_2_ sample after individual electric current pulse applications along the *a*-axis of WTe_2_ for *I*_p_ = $\pm$6.5 mA (**b**) and $\pm$8.5 mA (**d**). **e**,**f** Field-free electrical SOT measurements and the corresponding quantum imaging results for a series of current pulses (*I*_p_) with a magnitude of $\pm$6.5 mA applied along the *b*-axis of WTe_2_. The scale bar is 5 μm in all figures. The SOT-driven magnetic switching and quantum sensing measurements are performed at 260 K.

Achieving deterministic and nonvolatile magnetization control constitutes a key technical challenge for developing modern spintronic memory and computing applications^1,2^. Next, we utilize the self-integrated hBN wide-field quantum microscopy to evaluate crystallographic symmetry-controlled field-free (in)deterministic Fe_3_GaTe_2_ magnetic switching. Figure 4a shows the “step-like” variations of anomalous Hall signals of the Fe_3_GaTe_2_ sample in response to a train of positive and negative current pulses, *I*_p_ = $\pm$6.5 mA, applied along the *a*-axis of WTe_2_. Robust deterministic magnetization switching feature is highlighted in a series of wide-field quantum images recorded after individual current pulse applications (Fig. 4b). We can see that the positive and negative large current pulses reliably switch the Fe_3_GaTe_2_ magnetic domains between two deterministic states, from magnetization up to magnetization down or vice versa, in agreement with the “step-like” variations of anomalous Hall signals shown in the bottom panel of Fig. 4a. When *I*_p_ is increased to $\pm$8.5 mA, the magneto-transport results basically remain the same (Fig. 4c). However, a microscopic view provided by hBN quantum imaging indicates that the observed magnetic switching of Fe_3_GaTe_2_ has deviated from the perfect deterministic nature. A small portion of the Fe_3_GaTe_2_ magnetic domains cannot be switched reproducibly between the two magnetic easy states. Although field-free deterministic magnetic switching still dictates most of the Fe_3_GaTe_2_ magnetization, it is evident that some local magnetic “patches” show random variations after every electrical writing cycle [Fig. 4d]. The observed local stochastic magnetization switching feature is attributable to the current-induced Joule heating effect, which introduces random demagnetization-remagnetization at certain Fe_3_GaTe_2_ sample areas where the local transient temperature is driven above the Curie point under large current pulse applications.

The deterministic magnetization control disappears when *I*_p_ is applied along the *b*-axis of WTe_2_. Figure 4f presents the evolution of Fe_3_GaTe_2_ magnetic stray field maps in response to a positive and negative pulse current train (*I*_p_ = $\pm$6.5 mA) applied along the high-symmetry *b*-axis of WTe_2_. In this case, fully randomly oriented magnetic domains are spontaneously formed over the entire Fe_3_GaTe_2_ sample area with a net out-of-plane magnetization approximately zero due to the absence of out-of-plane spins from WTe_2_. While the measured anomalous Hall signals basically fluctuate around the zero value with little variations (bottom panel of Fig. 4e), hBN quantum imaging shows that the microscopic Fe_3_GaTe_2_ magnetic patterns have dramatically changed at the nanoscale during the stochastic magnetization process (see Supplementary Information Note 9 for details).

### Imaging electric current distributions in the all-vdW SOT system

**Fig. 5:**
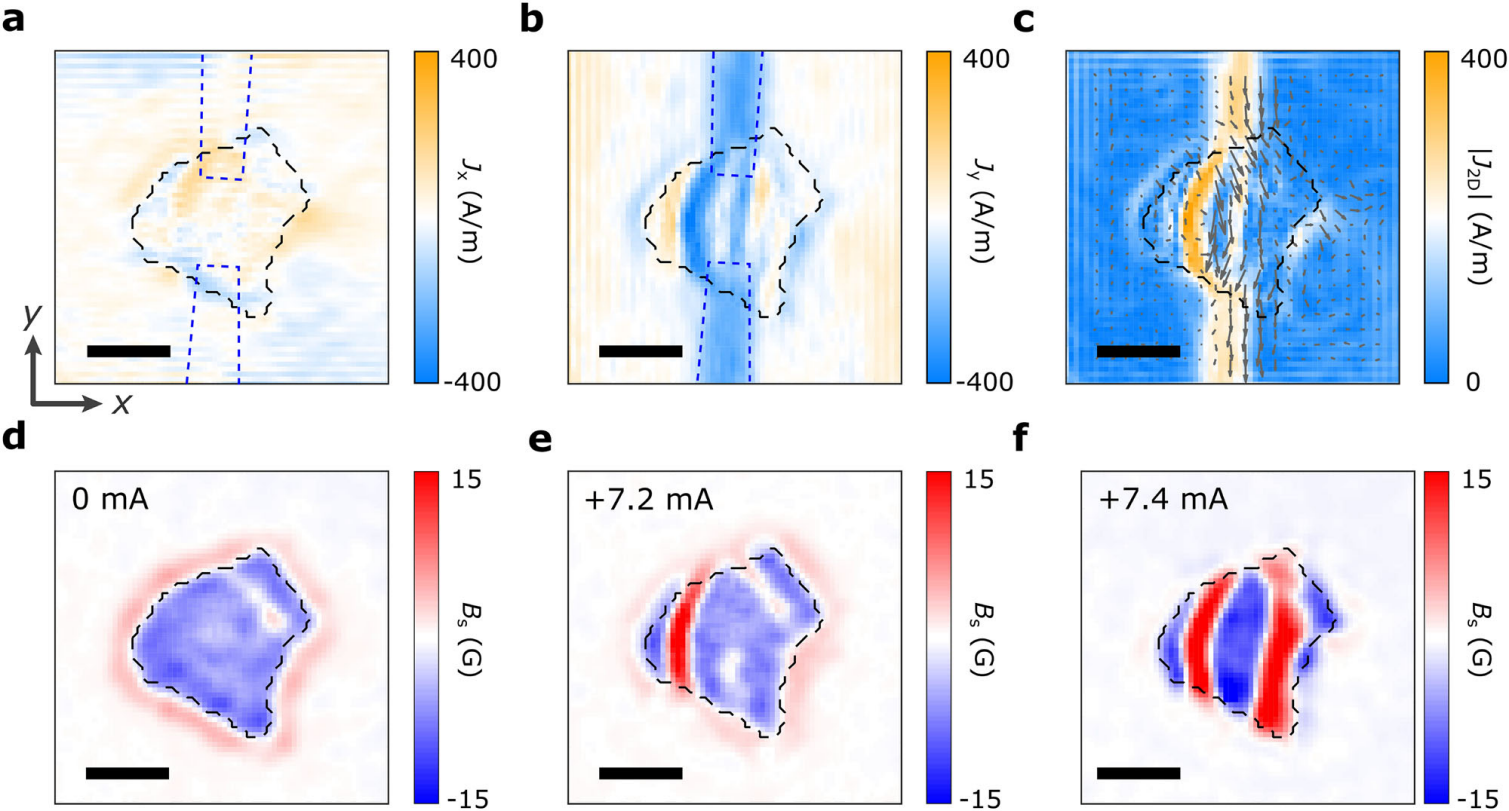
Quantum sensing of microscopic electric current flow distribution. **a**–**c** Spatially resolved transverse (*J*_*x*_), longitudinal (*J*_*y*_), and 2D electric current density ($\left|{J}_{2{{\rm{D}}}}\right|$) measured in a separately prepared WTe_2_/Fe_3_GaTe_2_/hBN device (device C). The input current between two Pt electrodes is 3.7 mA. Black and blue dashed lines outline the boundary of the Fe_3_GaTe_2_ flake and Pt electrodes for electric current application. **d–f** Evolution of unconventional SOT-driven field-free magnetic switching pattern (device C) when the electrical write current pulse increases from 0 mA to 7.2 mA and 7.4 mA. The scale bar is 3 μm in all figures.

Microscopic electric current distribution plays an important role in determining the magnetic switching performance and energy efficiency of SOT devices^1^. This point is particularly relevant for all-vdW SOT systems since their local current flow pathways are challenging to be precisely measure or predicted due to irregular sample shapes, potentially nontrivial topology, and vdW stacking effects. The demonstrated self-integrated quantum sensing platform provides an ideal solution to address this problem. Using the hBN wide-field quantum microscopy, spatially varying Oersted fields generated by local electric currents in the WTe_2_/Fe_3_GaTe_2_/hBN device are detected through optically detected magnetic resonance (ODMR) measurements (see Supplementary Information Note 3 for details). Invoking the Biot-Savart law, the 2D current flow pattern of the vdW heterostructure can be reconstructed as shown in Fig. 5a–c (see Supplementary Information Note 11 for details)^15^. We observe spatially nonuniform current distribution between two Pt electrodes of the WTe_2_/Fe_3_GaTe_2_/hBN device, where the charge current flow is largely confined in certain filament-like conduction channels in the low-current regime (Fig. 5c). We highlight that the measured microscopic current flow patterns are mutually corroborative with the spatially resolved SOT-driven magnetic switching behaviors as presented in Fig. 5d–f, allowing for selective control of local Fe_3_GaTe_2_ magnetization switching in current active areas. When an input electric current pulse *I*_p_ = 7.2 mA (Fig. 5e), Fe_3_GaTe_2_ magnetic moment is effectively switched from magnetization down to magnetization up in sample areas where the electric current is concentrated. Magnetic stray field in other device regions basically remains the same since the local current density has not reached the threshold value to trigger SOT switching. When further increasing *I*_p_ to 7.4 mA, the observed magnetic switching extends to the neighboring sample regions with high-density electric current flow, as shown in Fig. 5f. The ultimate magnetic switching ratio is co-determined by local current distributions, defects, inhomogeneities, pinning effect, and other material properties of the vdW device^1^. We also utilize partial coverage of WTe_2_ as an alternative approach to investigate the spatial control of SOT-driven magnetic switching of Fe_3_GaTe_2_. Details of these extended studies and simulation results can be found in Supplementary Information Note 12.

In summary, we have demonstrated a multimodal vdW heterostructure platform with dual quantum sensing and SOT functionalities. Unconventional SOT-driven nanoscale nucleation and evolution of magnetic domains in atomically thin Fe_3_GaTe_2_ are visualized by spin ensembles in the hBN encapsulation layer. We further reveal how the observed field-free magnetic switching evolves from a deterministic to a probabilistic nature due to the interplay between out-of-plane spins, in-plane spins, anisotropy, and Joule heating effects. Our wide-field imaging results show that the Fe_3_GaTe_2_ sample reliably switches between two deterministic magnetic states when current pulses are applied along the low-symmetry crystallographic axis of the spin source material WTe_2_. Micromagnetic simulations suggest the important role of field-like SOT in inhibiting thermal fluctuation-driven stochastic magnetization dynamics and chaotic multi-domain competition behaviors, thereby effectively assisting in the deterministic switching process. The deterministic nature of the field-free Fe_3_GaTe_2_ magnetic switching starts to degenerate when the magnitude of the electrical write current is above a certain threshold value due to the involvement of the local thermal heating effect. Spatially resolved electric current distribution in the vdW heterostructure is also imaged using the hBN quantum sensing techniques, and the current density-dependent SOT switching effect has been observed. Our study sheds light on the underlying mechanisms of unconventional SOT-driven magnetization dynamics, providing insights into future material design, integration, and engineering of vdW spintronic devices. The presented self-integrated hBN quantum sensing platform opens new opportunities for exploring nanoscale electromagnetic behaviors in a broad family of miniaturized 2D device systems^3,18,22,36^.

## Methods

### Material synthesis

The current study utilizes ^10^B-enriched monoisotopic hBN crystals synthesized by a metal (Ni-Cr) flux method^37^. The starting materials include 48 wt.% Ni (Alfa Aesar, 99.70%), 48 wt.% Cr (Alfa Aesar, 99.99%), and 4 wt.% ^10^B (Ceradyne 3M, 96.85%) powders. The source of elemental nitrogen is flowing N_2_ gas (ultra-high purity, 125 sccm), and 5 sccm H_2_ gas is used to minimize impurities originating from oxygen and carbon. ${V}_{{{\rm{B}}}}^{-}$ spin defects are created by thermal neutron irradiation^26^ of monoisotopic hBN crystals using a dose of ~2.6 × 10^16^ neutrons∙cm^−2^. Detailed information on preparing Fe_3_GaTe_2_ single crystals has been reported in previous literature^25,38^.

WTe_2_ crystals are synthesized via the self-flux method^39^. The starting materials [W powder (5N), Te lumps (5N)] with the initial composition W:Te = 1:49 are placed in a 2 mL Canfield Crucible Set^40^, and sealed in a fused silica ampoule under vacuum. The ampoule is heated up to 1000 °C, held for 10 h, and then slowly cooled down (2 °C/hour) to 525 °C. The single crystals are separated by centrifugation and then placed in another vacuum-sealed quartz tube and heated to 425 °C in a tube furnace for 2 days. The WTe_2_ crystals sit on the hot end, and the cold end is kept at room temperature. This step removes excess Te from the crystal surfaces via self-vapor transport and also anneals the crystals^41^.

### Device fabrication

WTe_2_/Fe_3_GaTe_2_/hBN vdW devices are prepared by the standard polydimethylsiloxane stamp process^42^. We first exfoliate bulk vdW crystals onto SiO_2_/Si substrates to obtain Fe_3_GaTe_2_, WTe_2_, and hBN nanoflakes. Selected flakes with desirable thicknesses and lateral dimensions are picked up by a poly (bisphenol A carbonate) stamp in sequence, then released onto SiO_2_/Si (285 nm) substrates with 7-nm-thick patterned Pt electrodes for transport measurements. The thicknesses of WTe_2_, Fe_3_GaTe_2_ and hBN flakes are deduced from optical contrast and confirmed by atomic force microscopy measurements. The 2D device fabrication processes are performed inside an argon-filled glovebox to minimize environmental effects. We have prepared multiple WTe_2_/Fe_3_GaTe_2_/hBN devices to ensure the consistency of the presented experimental results. The SOT and quantum imaging results measured on devices A and C are presented in the main text, and extended data of devices A, B, and C are included in the Supplementary Information. A Fe_3_GaTe_2_/hBN bilayer (device D) is used to characterize the magneto-transport properties of Fe_3_GaTe_2_ (see Supplementary Information Note 2 for details). Another WTe_2_/Fe_3_GaTe_2_/hBN device (device E) with a Fe_3_GaTe_2_ flake partially covered by a WTe_2_ flake is prepared to investigate spatial control of field-free magnetic switching (see Supplementary Information Note 12 for details). Separate samples are prepared for STEM and second-harmonic generation characterizations.

### SOT measurements

We use the standard four-probe method to measure the anomalous Hall response and to electrically detect field-free magnetic switching of Fe_3_GaTe_2_ in all prepared all-vdW SOT devices. An electrical write current pulse (*I*_p_) with a time duration of 100 µs is first applied in the current channel of a device. Anomalous Hall response of Fe_3_GaTe_2_ is measured by a sensing current of 0.1 mA after a time interval of 2 s. The magnitude of electrical write current pulses is systematically varied to investigate SOT effects on magnetic switching.

For measurements of the shift of anomalous Hall loops, we apply an electrical write current pulse (*I*_p_) with a duration of 300 µs, and the anomalous Hall signal of Fe_3_GaTe_2_ is detected with a read delay of 200 µs. The magnitude of electrical write current pulses (*I*_p_) is fixed during the anomalous Hall loop shift measurements.

### Wide-field quantum imaging measurements

The prepared WTe_2_/Fe_3_GaTe_2_/hBN devices are positioned in a closed-cycle optical cryostat for wide-field quantum magnetometry measurements. We apply continuous-wave (CW) green laser and microwave signals to perform ODMR measurements. We sweep the frequency *f* of CW microwave signals and measure the fluorescence of ${V}_{{{\rm{B}}}}^{-}$ spin defects across the field of view projected on a CMOS camera. The numerical aperture of the microscope objective used for our wide-field imaging is 0.75NA with a working distance of 4 mm. When *f* matches the electron spin resonance frequencies, ${V}_{{{\rm{B}}}}^{-}$ spin defects are excited to the m_s_ = $\pm$1 state(s), which are more likely to relax through a non-radiative pathway back to the m_s_ = 0 ground state and emit reduced PL. The laser power entering the optical cryostat is ~8 mW and the wide laser beam spot after passing the objective is ~20 $\times$ 20 μm. Microwave currents are generated by a Rohde & Schwarz signal generator and amplified by $+$50 dB before being sent to a gold wire suspended above the prepared vdW devices. The external magnetic field is zero during electric current pulse applications.

### Micromagnetic simulations

Micromagnetic simulations are conducted using an open-source, GPU-accelerated MUMAX3 package^43^. The simulation grid is dimensioned at 400 $\times$ 400 $\times$ 7 cells, with each sized at 5  5 $\times$ 1 nm^3^. Our simulations are based on the experimentally measured saturation magnetization value (*M*_s_ = 2.1 $\times$ 10^4 ^A/m) of Fe_3_GaTe_2_ at 260 K. The *M*_s_ value at 0 K used as a reference for scaling is extracted to be 4.0$\times$ 10^4 ^A/m. Other material parameters in our simulations include: exchange stiffness *A*_ex_ = 7.5 $\times$ 10^-12 ^J/m^44^, first-order uniaxial anisotropy *Ku*_1_ = 2.0 $\times$ 10^5 ^J/m^3^^44^, interfacial Dzyaloshinskii–Moriya interaction (DMI) strength *D*_dmi_ = 0.6 $\times$ 10^-3 ^J/m^2^^44^, Gilbert damping constant $\alpha$ = 0.005, and the effective charge-to-spin conversion efficiency of WTe_2_ ($\xi$ = 0.18). The exchange stiffness, first-order uniaxial anisotropy, and DMI interaction are considered to be phenomenologically temperature scaling with *M*_s_ (*T*) as follows: ${A}_{{ex}}\propto {M}_{s}^{2}\,\left(T\right)$, *Ku*_1_ $\propto {M}_{s}^{3}\,\left(T\right)$, and *D*_dmi_$\,\propto {M}_{s}^{2}\left(T\right)$. The Fe_3_GaTe_2_ magnetization dynamics are simulated by the Landau-Lifshitz-Gilbert-Slonczewski equation^45,46^:

$$\frac{\partial {{\boldsymbol{m}}}}{\partial t}=-\gamma {{\boldsymbol{m}}}\times {{{\boldsymbol{H}}}}_{{{\rm{eff}}}}+\alpha {{\boldsymbol{m}}}\times \frac{\partial {{\boldsymbol{m}}}}{\partial t}-\frac{\gamma {{\hslash }}\xi J}{2e{M}_{{{\rm{s}}}}t}{{\boldsymbol{m}}}\times \left({{\boldsymbol{m}}}\times {{\boldsymbol{\sigma }}}\right)-{\eta }_{{{\rm{FL}}}}\frac{\gamma {{\hslash }}\xi J}{2e{M}_{{{\rm{s}}}}t}{{\boldsymbol{m}}}\times {{\boldsymbol{\sigma }}}$$

where $\gamma$ is gyromagnetic ratio, $\hslash$ is the reduced Planck constant, *e* is the electron charge, ***H***_eff_ is the effective magnetic field of Fe_3_GaTe_2_, ${{\boldsymbol{\sigma }}}$ is the polarization of spin currents, *t* is the thickness of the Fe_3_GaTe_2_ sample, $\eta$_FL_ is the field-like torque efficiency, and *J* is the electric current density in WTe_2_. All simulations are initialized with the Fe_3_GaTe_2_ magnetization uniformly aligned along the positive *z*-axis direction. The temporal evolution of the magnetic configuration is then simulated. To ensure numerical accuracy of our micromagnetic model, the cell size is chosen to be significantly smaller than the exchange length, which is defined by ${l}_{{ex}}=\sqrt{{A}_{{ex}}/\left({{{\rm{\mu }}}}_{0}{M}_{s}^{2}\right)}$ (${{{\rm{\mu }}}}_{0}$ is the vacuum permeability). To reproduce the characteristic behaviors of the much larger real sample, we have also applied periodic boundary conditions to minimize finite-size-induced edge effects. External magnetic fields are absent in the presented simulation results.

### Atomic-resolution STEM measurements

Electron transparent samples for STEM measurements are prepared using Thermo Fisher Scientific (TFS) Helios 650 Nanolab focused ion beam (FIB) using the standard lift-out method: a 200-nm-thick carbon and 1-µm-thick Pt are deposited as a protective capping layer on the device; the samples are thinned with a final 2 kV Ga ion followed by 1 kV beam to mitigate any surface damage effects; the device is stored in an inert environment inside a glove box to prevent degradation.

HAADF-STEM is performed on an aberration-corrected TFS Spectra operated at 300 kV with a 30 mrad convergence angle. The inner and outer collection angles for HAADF are 60 and 200 mrad, respectively. The HAADF-STEM image presented in Fig. 1d is acquired as a series of 50 rapid-frame images (500 ns each) and subsequently realigned and averaged using the rigid registration method^47^ to produce a high-fidelity and high signal-to-noise image of the atomic lattice.

## Supplementary information

Supplementary Information

Description of Additional Supplementary Files

Supplementary Video 1

Transparent Peer Review file

## Source data

Source Data

## Data Availability

All data supporting the findings of this study are available from the corresponding authors on reasonable request. Source data are provided with this paper.
