# Supplementary material for: Imaging of a van der Waals spin-orbit torque system using spin ensembles in hBN
Source: Nat Commun. 2026 Jun 10;17:7350. doi: 10.1038/s41467-026-74178-7 (PMC13402656; doi:10.1038/s41467-026-74178-7)
Supplement: Supplementary file 1 — Supplementary Information [file 41467_2026_74178_MOESM1_ESM.pdf]

Supplementary Information for
Imaging of a van der Waals Spin-Orbit Torque System Using Spin Ensembles in hBN

Xi Zhang[1,+], Jingcheng Zhou[1,+], Chaowei Hu[2], PeiYu Cai[3], Kuangyin Deng[4], Chuangtang Wang[5], Nishkarsh Agarwal[6], Hanshang Jin[7], Faris A. Al-Matouq[1], Stelo Xu[7], Roshan S. Trivedi[1], Senlei Li[1], Sumedh Rathi[1], Hanyi Lu[8,1], Zhigang Jiang[1], Valentin Taufour[7], Robert Hovden[6,9], Liuyan Zhao[5], Ran Cheng[4,10], Xiaodong Xu[2,11], Elton J. G. Santos[3,12,13], Jiun-Haw Chu[2], Chunhui Rita Du[1,*], and Hailong Wang[1,*]

[1]School of Physics, Georgia Institute of Technology, Atlanta, Georgia 30332, USA
[2]Department of Physics, University of Washington, Seattle, Washington 98105, USA
[3]Institute for Condensed Matter and Complex Systems, School of Physics and Astronomy, University of Edinburgh, Edinburgh EH9 3FD, UK
[4]Department of Electrical and Computer Engineering, University of California, Riverside, California 92521, USA
[5]Department of Physics, University of Michigan, Ann Arbor, Michigan 48109, USA
[6]Department of Materials Science and Engineering, University of Michigan, Ann Arbor, Michigan 48109, USA
[7]Department of Physics and Astronomy, University of California, Davis, California 95616, USA
[8]Department of Physics, University of California, San Diego, La Jolla, California 92093, USA
[9]Applied Physics Program, University of Michigan, Ann Arbor, Michigan 48109, USA
[10]Department of Physics and Astronomy, University of California, Riverside, California 92521, USA
[11]Department of Materials Science and Engineering, University of Washington, Seattle, Washington 98105, USA
[12]Higgs Centre for Theoretical Physics, University of Edinburgh, Edinburgh EH9 3FD, UK.
[13]Donostia International Physics Center, Donostia-San Sebastián 20018, Spain.

[*]Corresponding authors: cdu71@gatech.edu, hwang3021@gatech.edu
[+]These authors contributed equally.

Supplementary Note 1. Thickness characterization of prepared van der Waals devices

Van der Waals (vdW) heterostructures in the current study are composed of hexagonal boron nitride (hBN) layers containing boron vacancy V_B^- spin defects, Fe_3GaTe_2 and WTe_2 nanoflakes artificially stacked with each other in sequence by standard polydimethylsiloxane stamp processes[1]. Supplementary Figs. 1a-1c present optical images of three WTe_2/Fe_3GaTe_2/hBN vdW devices (devices A, B, and C) for wide-field quantum imaging and electrical transport measurements. Thicknesses of individual vdW layers are estimated by optical contrast and confirmed by atomic force microscopy (AFM) measurements. Note that scanning directions of the AFM tip are set to be perpendicular to all steps to ensure accuracy of thickness characterizations. The thicknesses of the hBN, WTe_2 and Fe_3GaTe_2 flakes are measured to be approximately 65 nm, 13.8 nm, and 6.3 nm in device A, 67 nm, 9.6 nm, and 7.2 nm in device B, and 32 nm, 10.7 nm, and 7.1 nm in device C as shown in Supplementary Figs. 1e-1g. We also prepare device D with a 6.9-nm-thick Fe_3GaTe_2 flake encapsulated by a hBN flake on SiO_2/Si (285 nm) substrate(s) with prepatterned Pt electrodes. Device D is used for characterizing magneto-transport properties of Fe_3GaTe_2 as discussed in the section below. Supplementary Figs. 1d and 1h present the optical image of device D and the corresponding AFM characterization of constituent vdW layer thicknesses. WTe_2/Fe_3GaTe_2/hBN device E with a Fe_3GaTe_2 flake partially covered by a WTe_2 flake is fabricated to investigate spatial control of SOT-driven magnetic switching in Fe_3GaTe_2 (see Supplementary Information Note 12 for details).

Supplementary Note 2. Magneto-transport characterizations of Fe_3GaTe_2 nanoflake(s)

We utilize device D to characterize magneto-transport response of an atomically thin Fe_3GaTe_2 flake. Supplementary Fig. 2a presents anomalous Hall resistance (R_{AHE}) of the Fe_3GaTe_2 sample (~6.9 nm) measured as a function of a perpendicular magnetic field in the temperature range from 5 K to 310 K. One can see that the anomalous Hall hysteresis loops show the characteristic square shape below 270 K, and the magnetic coercive field H_c monotonically decreases with increasing temperature. Above 270 K, the measured Hall hysteresis loops become tilted due to enhanced thermal fluctuations and reduced perpendicular magnetic anisotropy. The anomalous Hall feature disappears above 310 K. Supplementary Figs. 2b and 2c summarize the temperature dependences of R_{AHE} and H_c of the Fe_3GaTe_2 sample. The Curie temperature is estimated to be 310 K, consistent with our wide-field quantum imaging results.

Supplementary Note 3. Optically detected magnetic resonance (ODMR) sensing using V_B^- spin defects

A negatively charged V_B^- spin defect in hBN is formed by a boron vacancy and three surrounding nitrogen atoms. It has a spin triplet ground state ($m_s = 0, \pm1$) with a zero-field splitting of ~3.5 GHz at room temperature. Supplementary Fig. 3a shows the V_B^- spin energy level as a function of a magnetic field B applied along the V_B^- spin axis (out-of-plane direction). When $B > 0$, the linear Zeeman effect separates the $m_s = -1$ and $m_s = +1$ V_B^- spin state and the magnitude of B can be extracted as follows[2]:

$$B = \frac{\pi\sqrt{(f_+ - f_-)^2 - 4E_0{}^2}}{\tilde{\gamma}} \tag{1}$$

where $f_\pm$ denote the electron spin resonance (ESR) frequencies of V_B^- spin transitions between the m_s = 0 and m_s = ± 1 states, E_0 is the off-axial zero-field splitting parameter, and γ is the gyromagnetic ratio of V_B^- centers. The "three-level" V_B^- spin system can be optically accessed by spin-dependent photoluminescence (PL) in ODMR measurements. Under green laser excitation, a V_B^- center excited to the triplet excited state decays back to the ground state by emitting near-infrared fluorescence. Meanwhile, a metastable V_B^- spin state also exists through which the m_s = ± 1 excited states are more likely to relax non-radiatively back to the m_s = 0 ground state, generating reduced PL. Supplementary Fig. 3b shows a typical ODMR spectra of V_B^- spin ensembles measured with an out-of-plane oriented magnetic field B = 50 G at 260 K. Using the above equation, the magnitude of B can be quantitatively obtained in ODMR sensing measurements.

Supplementary Note 4. Examination of local crystal symmetry of 2D WTe_2 flakes

The noncentrosymmetric crystal structure of WTe_2 is evaluated by rotational anisotropy second harmonic generation (RA-SHG) measurements[3–7]. The incident light, with a wavelength centered at 800 nm, has a pulse duration of 80 fs and a repetition rate of 200 kHz. A 20x objective is employed to focus the light on a spot size of ~3 μm under normal incidence. The reflected SHG light is collected by the same objective then directed into an electron-multiplying charge-coupled device for intensity measurements. The polarizations of incident and reflected lights are selected to be parallel. The obtained RA-SHG pattern is fitted based on the *m* point group under the electric dipole response[7]: $I_{parallel}^{2\omega}(\phi) = \left(\chi_{yyy}^{ED}\cos^3(\phi) + \left(2\chi_{yxy}^{ED} + \chi_{yxx}^{ED}\right)\sin^2(\phi)\cos(\phi)\right)^2$, where $I_{parallel}^{2\omega}(\phi)$ is the SHG intensity in the parallel channel, ϕ is the azimuthal angle of the polarization with respect to the *a*-axis of WTe_2, and χ_{yyy}^{ED}, χ_{yxy}^{ED} and χ_{yxx}^{ED} are the nonzero susceptibility tensors in the normal incidence condition. A typical RA-SHG polar plot of a few-layer-thick WTe_2 flake is presented in Supplementary Fig. 4, from which we can identify the *bc* mirror plane of WTe_2 with zero SHG intensity, and hence, the crystallographic *a*-axis that is normal to this mirror plane[7].

Supplementary Note 5. Extended spin-orbit torque measurements

We have performed extended spin-orbit torque (SOT) measurements to investigate (in)deterministic magnetic switching in WTe_2/Fe_3GaTe_2/hBN vdW devices over a broad range of temperatures. Supplementary Fig. 5 presents a series of current driven anomalous Hall loops of the Fe_3GaTe_2 sample recorded at temperatures ranging from 170 K to 260 K. Electric current pulse(s) I_p is applied along the low-symmetry crystallographic axis (*a*-axis) of WTe_2 in absence of an external auxiliary magnetic field. Robust field-free deterministic switching is observed at all the measurement temperatures. It is worth noting that the threshold current for magnetic switching gradually decreases with increasing temperatures due to enhanced thermal fluctuations that help the Fe_3GaTe_2 magnetization overcome the potential barrier between two magnetic easy states. Supplementary Fig. 6 shows the field-free SOT results measured at temperatures from 180 K to 250 K when I_p flows along the high-symmetry crystallographic axis (*b*-axis) of WTe_2. It is evident that the signature of deterministic magnetic switching vanishes in this situation due to the lack of symmetry breaking induced out-of-plane spins from WTe_2. The Fe_3GaTe_2 sample enters an intermediate magnetic phase with nearly compensated net out-of-plane magnetization under large

positive and negative current pulse applications. The extended measurement results corroborate the symmetry-dependent SOT magnetization control by spin currents from WTe_2.

Supplementary Note 6. Characterizations of unconventional SOT efficiency of WTe_2

In this section, we provide the details to estimate the SOT efficiency of out-of-plane spin currents generated by WTe_2. We start from characterizing the out-of-plane effective SOT field ΔH_{oop} produced by the spin source material. Supplementary Figs. 7a-7d present anomalous Hall response of the Fe_3GaTe_2 sample under positive and negative I_p with different magnitudes flowing along the a-axis direction of WTe_2. Clear shift of anomalous Hall loops is observed when the magnitude of current pulse(s) $|I_P|$ is above 6 mA (Supplementary Figs. 7c and 7d). Supplementary Fig. 7e summarizes ΔH_{oop} measured as a function of $|I_P|$. Here, we define the shift of anomalous Hall loop(s) as: ΔH_{oop} (I_p) = $[H_c^+(I_p) + H_c^-(I_p)]/2$, where $H_c^+(I_p)$ and $H_c^-(I_p)$ are the positive and negative magnetic coercive fields of the Fe_3GaTe_2 sample under electric current pulse application, respectively. When $|I_P|$ is smaller than the critical value, ΔH_{oop} is close to zero as shown in Supplementary Figs. 7a and 7b. One can see that the measured ΔH_{oop} shows a characteristic threshold current effect, in agreement with the physical picture that out-of-plane antidamping torque generated from WTe_2 overcomes the intrinsic magnetic damping of Fe_3GaTe_2 above a critical electric current application. The SOT field per unit current density is given by $\chi = \Delta H_{oop}/J$, where J is the electric current density in the spin source material. The effective SOT efficiency ξ of out-of-plane spin currents from WTe_2 can be calculated by the following equation[8–10]:

$$\xi = \frac{2e\mu_0 M_s t}{\hbar}\chi \qquad (2)$$

where $\hbar$ is the reduced Planck constant, e is the electron charge, μ_0 is the vacuum permeability, M_s and t are the saturation magnetization and thickness of the Fe_3GaTe_2 sample, respectively. Using the value of I_p close to the critical electrical switching current (I_p = 6.14 mA), ξ is estimated to be 0.180 ± 0.045 in the current study. Based on the transport measurement in the previous work[11,12], the electrical resistivities of Fe_3GaTe_2 and WTe_2 flakes are used to be (0.155 ± 0.015) mΩ·cm and (1.000 ± 0.200) mΩ·cm in our calculations.

We also investigate the out-of-plane effective SOT field (ΔH_{oop}) generated in the prepared vdW stacking device when I_p is applied along the high-symmetry axis of WTe_2 as shown in Supplementary Figs. 7f-7i. In this case, ΔH_{oop} shows a weak current dependence and random oscillations around zero as summarized in Supplementary Fig. 7j, indicating a negligible out-of-plane antidamping SOT effect due to the preserved mirror symmetry in the bc-plane of WTe_2.

Supplementary Note 7. hBN-quantum imaging of magnetic phase transition in atomically thin Fe_3GaTe_2

We utilize hBN-based wide-field quantum imaging techniques to investigate the magnetic phase transition in Fe_3GaTe_2 flake(s) in the current work. As discussed in the Supplementary Note 3 above, an out-of-plane oriented magnetic field B at local V_B^- sites can be measured by the ODMR sensing method. In our experiments, B has contributions from both the external magnetic field B_{ext} along z-axis direction and the z-component of magnetic static stray field B_s emanating from the Fe_3GaTe_2 sample. By subtracting B_{ext}, stray field B_s exclusively generated by the Fe_3GaTe_2 flake

can be obtained. By performing spatially dependent ODMR measurements over V_B^- spin ensembles, we can obtain a 2D stray field map as shown in Fig. 1c in the main text.

Next, we introduce the method to reconstruct 2D Fe_3GaTe_2 magnetization from the measured magnetic stray field map. Supplementary Fig. 8 shows the coordinate system used for our numerical analysis. hBN and Fe_3GaTe_2 flakes lie in the *x*-*y* plane. The Fe_3GaTe_2 sample with a thickness of t_{FGT} occupies the space of $-t_{FGT} \leq z \leq 0$ and V_B^- spin defects are arranged on individual hBN atomic layers. Thickness of the hBN flake is h_2. In the global frame, the stray field distribution $\mathbf{B}_s(\mathbf{R})$ is related to the magnetization distribution $\mathbf{M}(\mathbf{R}')$ of the Fe_3GaTe_2 sample in the following way[13]:

$$\mathbf{B}_\mathrm{s}(\mathbf{R}) = \int \mathrm{d}^3\mathbf{R}' D\left(\mathbf{R}, \mathbf{R}'\right) \mathbf{M}\left(\mathbf{R}'\right) \tag{3}$$

where $D\left(\mathbf{R}, \mathbf{R}'\right) = -\nabla_\mathrm{R}\nabla_{\mathrm{R}'}\left(1/\left|\mathbf{R} - \mathbf{R}'\right|\right)$ is the magnetostatic Green's function tensor between coordinates $\mathbf{R} = (x, y, z)$ and $\mathbf{R}' = (x', y', z')$. We take the 2D Fourier transform for Eq. (3), and when the translational symmetries are present, magnetic stray field $\mathbf{B}_s$ at positions of V_B^- defects can be written as:

$$\mathbf{B}_\mathrm{s}(\mathbf{k}) = \int D\left(\mathbf{k}, z'\right) \mathbf{M}(\mathbf{k})\mathrm{d}z' \tag{4}$$

where $\mathbf{M}(\mathbf{k})$ is the effective 2D magnetization distribution function of the Fe_3GaTe_2 sample in the Fourier space and $\mathbf{k} = \left(k_x, k_y, 0\right)$. Due to the out-of-plane anisotropy, the Fe_3GaTe_2 magnetization is spontaneously ordered perpendicular to the sample surface: $\mathbf{M}(\mathbf{k}) = M(\mathbf{k})\hat{\mathbf{z}}$. Thus, the *z*-component of magnetic stray field $B_s(\mathbf{k})$ can be expressed as follows:

$$\begin{aligned} B_\mathrm{s}(\mathbf{k}) &= \int_{h_1}^{h_2} D_{zz}\left(\boldsymbol{k}, z'\right) M(\boldsymbol{k})\mathrm{d}z' \\ &= 2\pi[e^{h_2 k} - e^{h_1 k}]M(\boldsymbol{k}) \end{aligned} \tag{5}$$

where $D_{zz}\left(\mathbf{k}, z'\right) = 2\pi k \exp\left(kz'\right)$ is the element of the Green's function tensor in Fourier space, which relates the *z*-component of $\mathbf{M}(\mathbf{k})$ to the *z*-component of $\mathbf{B}_s(\mathbf{k})$. Considering the magnetic quenching effect resulting from the exchange interaction of Fe_3GaTe_2 moments, our model has assumed that V_B^- spin defects are active for field sensing only when they are at least h_1 = 10 nm away from the Fe_3GaTe_2/hBN interface[14]. Lastly, the real space magnetization pattern can be reconstructed by introducing an inverse Fourier transform:

$$M(\mathbf{R}) = M(x, y) = \frac{1}{(2\pi)^2}\int M(\mathbf{k})e^{-i(k_x x + k_y y)}\mathrm{d}k_x\mathrm{d}k_y \tag{6}$$

We now present hBN-based quantum sensing of magnetic phase transition of Fe_3GaTe_2 nanoflakes. Supplementary Figs. 9a-9g present stray field maps of the Fe_3GaTe_2 sample (device A) measured at a series of temperatures between 40 K to 290 K. Using the method presented above, the corresponding Fe_3GaTe_2 magnetization pattern can be reconstructed as shown in Supplementary Figs. 10a-10g. In the low temperature regime ($T \leq 240$ K), the exfoliated Fe_3GaTe_2 flake exhibits robust magnetization, indicating a long-range magnetic order sustained by the intrinsic magnetocrystalline anisotropy. The measured Fe_3GaTe_2 magnetization decreases with increasing temperatures due to enhanced thermal fluctuations that tend to perturbate the spontaneous magnetic order. When approaching the Curie temperature where the thermal fluctuation energy becomes comparable with that of the local magnetic exchange interaction, we

observe a significant reduction of the sample magnetization all the way down to zero, indicating a ferromagnet-paramagnet phase transition in Fe_3GaTe_2. Above the Curie point, emanating stray fields virtually vanish and the measured (ferro)magnetization completely disappears in the Fe_3GaTe_2 sample. Supplementary Figs. 9h and 10h summarize the temperature dependence of the stray field (B_s) and magnetization ($4\pi M_s$) measured at a local sample site of the Fe_3GaTe_2 flake, which is highlighted by a black point in Supplementary Figs. 9a and 10a. One can see that B_s and $4\pi M_s$ exhibit a gradual decay in the low temperature regime, followed by a dramatic decrease during the magnetic phase transition of Fe_3GaTe_2 from which the Curie temperature is estimated to be ~310 K.

Supplementary Note 8. Extended wide-field quantum imaging results on device A

We now present extended wide-field quantum imaging results on field-free deterministic and probabilistic magnetic switching of device A. Supplementary Figs. 11a-11d show variations of the Fe_3GaTe_2 (device A) stray field maps in response to a series of electrical write current pulses $I_p = \pm$ 8.5 mA applied along the *a*-axis of WTe_2. It is evident that the Fe_3GaTe_2 magnetization shows bipolar switching depending on the polarity of electrical write current pulses. The magnetic switching ratio is estimated to be ~70 %, in qualitative agreement with our electrical SOT measurement results. While the deterministic switching feature dominates most of the sample areas, it is also noticed that a small portion of the Fe_3GaTe_2 magnetization cannot be switched in a reproducible way possibly due to enhanced local thermal heating in the high current regime (I_p = $\pm$ 8.5 mA). When $I_p = \pm$ 6.5 mA flows along the *b*-axis of WTe_2 in Supplementary Figs. 11e-11h, we again observe randomly oriented magnetic domains formed over the entire Fe_3GaTe_2 flake and the net perpendicular magnetization is close to zero due to the lack of out-of-plane spin currents or an auxiliary in-plane magnetic field to achieve deterministic magnetic control. The extended hBN imaging data are consistent with the results presented in Fig. 4 in the main text, corroborating the deterministic and stochastic nature of Fe_3GaTe_2 switching under different SOT conditions.

Supplementary Note 9. Wide-field imaging of field-free magnetic switching observed in devices B and C

We further perform hBN-based quantum imaging of field-free magnetic switching in WTe_2/Fe_3GaTe_2/hBN devices B and C. The quantum sensing measurements follow the same procedure as described in the manuscript. Electric current pulses (I_p) are applied along the *a*-axis of WTe_2 to achieve field-free deterministic magnetization control. Supplementary Figs. 12i and 13i present two SOT-driven magnetic hysteresis loops of devices B and C measured in absence of an external magnetic field. Supplementary Figs. 12a-12h and Figs. 13a-13h plot stray field maps of the Fe_3GaTe_2 flakes recorded at the corresponding points ("A" to "H") on the magnetic switching loops. Overall, the quantum imaging results measured on devices B and C share the similar field-free deterministic switching feature as observed in device A. It is instructive to note that the switchable magnetic domains by SOT in device B are not continuous, which is possibly due to magnetic inhomogeneities/defects creating local energy barriers to inhibit domain wall propagations in the Fe_3GaTe_2 sample. For device C, the magnetic switching ratio reaches above 90 % as highlighted by our quantum imaging results. The enhanced switching ratio observed in device C could be related to reduced sample dimensions, which is beneficial for achieving a more uniform magnetization control in vdW SOT devices.

Supplementary Figs. 14a-14d show variations of magnetic stray field patterns of device C in response to individual electric current pulse applications ($I_P = \pm 7.8$ mA) along the *a*-axis of WTe_2, confirming the field-free deterministic magnetic switching dictated by out-of-plane spin currents generated from WTe_2.

Supplementary Note 10. Simulations of stochastic magnetic switching of Fe_3GaTe_2 in absence of field-like SOT

In the main text, we have reported micromagnetic simulation results demonstrating that the perpendicular Fe_3GaTe_2 magnetization shows field-free deterministic switching under robust field-like SOT with $\eta_{FL} = 0.5$. Here, we present extended simulations to illustrate the transition from deterministic to stochastic, a far more chaotic switching dynamics when the field-like SOT is absent. Supplementary Fig. 15 plots the simulation of stochastic magnetic switching of Fe_3GaTe_2 with an electric current density $J = 2.6 \times 10^{11}$ A/m^2 in WTe_2 (along the *a*-axis) and $\eta_{FL} = 0$. Without the field-like SOT contribution, the Fe_3GaTe_2 magnetization reversal process is fundamentally less stable and fails to reach a fully switched state. It is noticed that the Fe_3GaTe_2 moment relaxes back to its original state after the electric pulse application. The difference in the switching mechanisms/results (relative to the case of $\eta_{FL} = 0.5$ presented in the main text) can be attributed to the role of field-like SOT in shaping the energy landscape of Fe_3GaTe_2. In contrast to the steady domain wall nucleation and propagation, the simulated magnetic switching proceeds via the stochastic nucleation of many small, competing domains throughout the bulk of the Fe_3GaTe_2 sample. These domains struggle against each other, leading to a noisy and fluctuating spatially averaged magnetization that fails to reach a fully switched state. A signature of this incoherent switching process (in absence of field-like SOT) is the necessity of a long post-pulse relaxation period, which involves slow domain merging and annihilation before the system eventually settles into the final magnetic equilibrium state. Our simulations suggest that a moderately strong ($\eta_{FL} > 0.2$) field-like SOT can effectively assist in driving domain wall movement swiftly and coherently across the Fe_3GaTe_2 sample, helping establish the "deterministic" nature of the observed field-free switching process.

Supplementary Note 11. Quantum sensing of electric current distributions in device C

We utilize the presented hBN wide-field quantum microscopy to image electric current flow in WTe_2/Fe_3GaTe_2/hBN device C. The exact measurement mechanism follows a similar way to the ODMR magnetometry as discussed above. The magnitude of Oersted fields generated by local charge currents is deduced from the splitting of spin energy levels of boron vacancy V_B^- defects in the hBN encapsulation layer[15]. Here, we individually measured the field maps with positive and negative electric current applications (at the same current magnitude) to exclude the contribution of magnetic stray fields from the Fe_3GaTe_2 magnetization. Note that Oersted fields generated by electric currents reverse their polarity accordingly while stray fields emanating from Fe_3GaTe_2 magnetization are independent on the current direction. By subtracting the contribution of magnetic stray fields, we can obtain the Oersted field map exclusively produced by electric currents as shown in Supplementary Fig. 16a. Using the inverse Biot-Savart law[15], spatially resolved current distribution is reconstructed as presented in Supplementary Fig. 16b.

Supplementary Note 12. Spatial control of SOT-driven magnetic switching of Fe_3GaTe_2

We have prepared a separate WTe_2/Fe_3GaTe_2/hBN device (device E) where a Fe_3GaTe_2 flake is partially covered by a WTe_2 flake to investigate spatial control of field-free magnetic switching. Supplementary Fig. 17a shows the optical microscopy image of device E. We first perform hBN quantum microscopy measurements to demonstrate SOT-driven selective control of Fe_3GaTe_2 magnetization. Supplementary Figs. 17b-17e present a series of wide-field quantum images recorded after individual electric current pulse applications ($I_p = \pm$ 8 mA along the *a*-axis of WTe_2). One can see that positive and negative current pulses partially switch local Fe_3GaTe_2 magnetic "patches" covered by WTe_2 from magnetization up to magnetization down or vice versa. In contrast, the stray field map basically remained the same in the Fe_3GaTe_2 sample area without the WTe_2 coverage (except for a few tiny edge areas of the sample).

We also simulate a device with a similar structure to theoretically study the spatial control of magnetic switching by the WTe_2 coverage. Our model consists of a Fe_3GaTe_2 sample where only the top half is capped with WTe_2. In this case, the SOT required to drive perpendicular magnetization switching, in principle, only presents in the Fe_3GaTe_2/WTe_2 region. Supplementary Figs. 17f-17j present simulated variations of Fe_3GaTe_2 magnetic domains in response to a series of electric current pulses applications ($J = \pm 1.5 \times 10^{11}$ A/m^2 along the *a*-axis of WTe_2). It is clear that the magnetization reversal is confined in the SOT-active region. Moreover, our simulations reveal that the switched domain could potentially extend slightly into the adjacent Fe_3GaTe_2 region uncapped with WTe_2, indicating that local exchange interaction can be strong enough to drag neighboring spins across the boundary.

Lastly, we check the potential effects of sample size and shape in our micromagnetic simulation results. We perform extended simulations across multiple system dimensions: 500×500 nm^2, 1000×1000 nm^2, and 2000×2000 nm^2 under a current density $J = 2.0 \times 10^{11}$ A/m^2 as shown in Supplementary Fig. 18. The switching trajectories are largely indistinguishable, suggesting that finite-size effects are negligible in the micrometer-scale regime and the SOT switching efficiency is mainly governed by the intrinsic nucleation barrier instead of the total sample area. Supplementary Fig. 19 presents our micromagnetic simulation results on a circularly shaped sample. One can see that the results (qualitatively) agree with those simulated on a square sample reported in the main text. Thus, we are convinced that our micromagnetic simulation model is sound and valid, which rationalizes well the experimental results.

References

1. Purdie, D. G. *et al.* Cleaning interfaces in layered materials heterostructures. *Nat. Commun.* **9**, 5387 (2018).
2. Huang, M. *et al.* Wide field imaging of van der Waals ferromagnet Fe_3GeTe_2 by spin defects in hexagonal boron nitride. *Nat. Commun.* **13**, 5369 (2022).
3. Jin, W. *et al.* Observation of a ferro-rotational order coupled with second-order nonlinear optical fields. *Nat. Phys.* **16**, 42–46 (2020).
4. Luo, X. *et al.* Ultrafast Modulations and Detection of a Ferro-Rotational Charge Density Wave Using Time-Resolved Electric Quadrupole Second Harmonic Generation. *Phys. Rev. Lett.* **127**, 126401 (2021).
5. Ahn, Y. *et al.* Electric quadrupole second-harmonic generation revealing dual magnetic orders in a magnetic Weyl semimetal. *Nat. Photonics* **18**, 26–31 (2024).
6. Guo, X. *et al.* Extraordinary phase transition revealed in a van der Waals antiferromagnet. *Nat. Commun.* **15**, 6472 (2024).
7. Drueke, E., Yang, J. & Zhao, L. Observation of strong and anisotropic nonlinear optical effects through polarization-resolved optical spectroscopy in the type-II Weyl semimetal T_d-WTe_2. *Phys. Rev. B* **104**, 064304 (2021).
8. Hu, S. *et al.* Efficient perpendicular magnetization switching by a magnetic spin Hall effect in a noncollinear antiferromagnet. *Nat. Commun.* **13**, 4447 (2022).
9. Wang, H. *et al.* Spin-Orbit-Torque Switching Mediated by an Antiferromagnetic Insulator. *Phys. Rev. Appl.* **11**, 044070 (2019).
10. Liu, L. *et al.* Spin-Torque Switching with the Giant Spin Hall Effect of Tantalum. *Science* **336**, 555–558 (2012).
11. Wu, S. *et al.* Robust ferromagnetism in wafer-scale Fe_3GaTe_2 above room-temperature. *Nat. Commun.* **15**, 10765 (2024).
12. Mleczko, M. J. *et al.* High Current Density and Low Thermal Conductivity of Atomically Thin Semimetallic WTe_2. *ACS Nano* **10**, 7507–7514 (2016).
13. van der Sar, T., Casola, F., Walsworth, R. & Yacoby, A. Nanometre-scale probing of spin waves using single electron spins. *Nat. Commun.* **6**, 7886 (2015).
14. Scholten, S. C. *et al.* Multi-species optically addressable spin defects in a van der Waals material. *Nat. Commun.* **15**, 6727 (2024).
15. Healey, A. J. *et al.* Quantum microscopy with van der Waals heterostructures. *Nat. Phys.* **19**, 87–91 (2023).

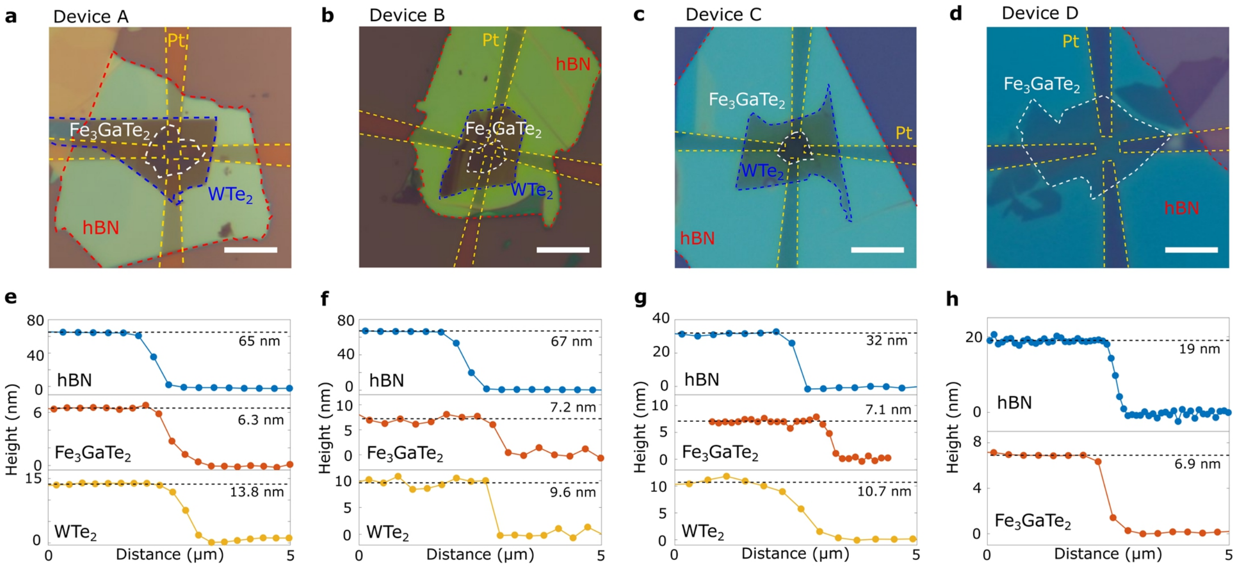

Supplementary Fig. 1. Thickness characterization of vdW flakes in prepared 2D devices. a-c, Optical microscopy images of prepared WTe_2/Fe_3GaTe_2/hBN device A (**a**), device B (**b**), and device C (**c**). **d**, Optical microscopy image of Fe_3GaTe_2/hBN device D used for characterizing temperature dependent magneto-transport properties of Fe_3GaTe_2. Boundaries of constituent vdW layers are outlined by red (hBN), blue (WTe_2), and white (Fe_3GaTe_2) dashed lines in **a-d**. Pt electrodes are highlighted by yellow dashed lines. The scale bar is 10 μm. **e-h**, One-dimensional AFM scans of WTe_2, Fe_3GaTe_2, and hBN flakes characterizing thicknesses of individual vdW layers in device A (**e**), device B (**f**), device C (**g**), and device D (**h**).

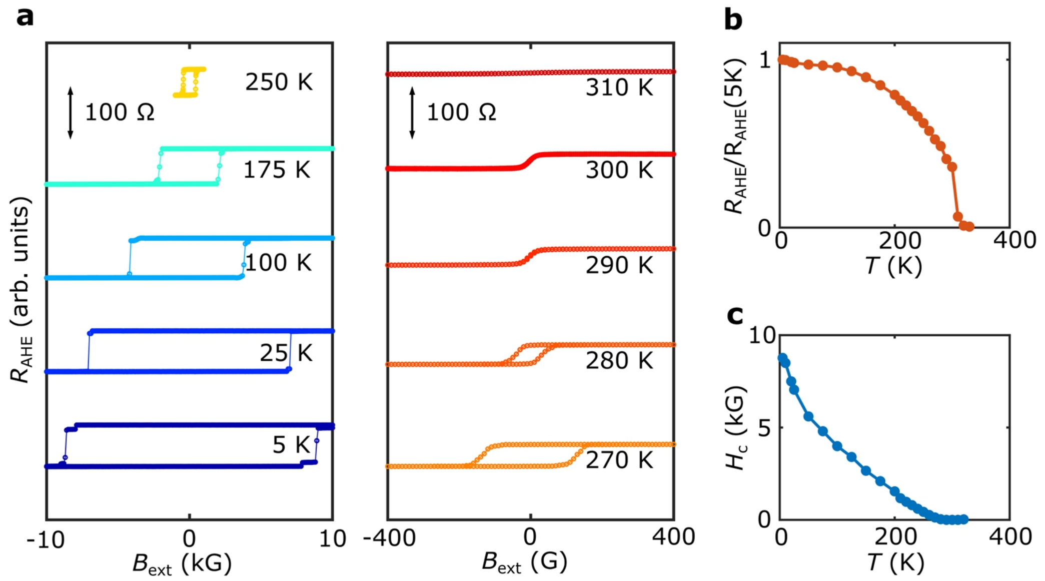

Supplementary Fig. 2. Magneto-transport characterization of atomically thin Fe_3GaTe_2. a, Anomalous Hall hysteresis loops of a ~6.9-nm-thick Fe_3GaTe_2 sample measured from 5 K to 310 K. **b-c**, Temperature dependences of normalized anomalous Hall resistance R_{AHE} (relative to R_{AHE} measured at 5 K) and magnetic coercive field (H_c) of the Fe_3GaTe_2 sample.

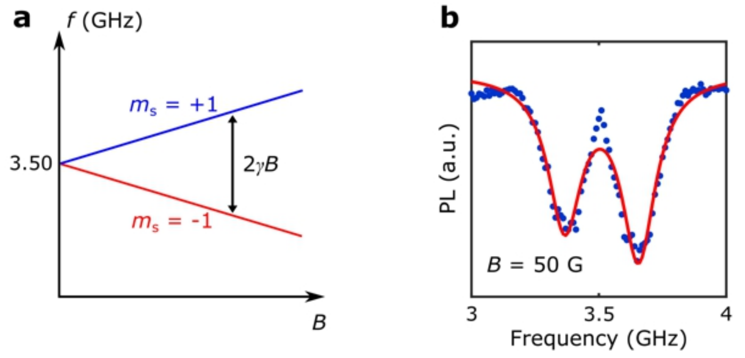

Supplementary Fig. 3. ESR measurements of V_B^- spin ensembles. a, V_B^- ESR frequencies as a function of magnetic field B along the V_B^- spin axis direction. **b**, A typical ODMR spectra of V_B^- spin defects measured with an external magnetic field B = 50 G at 260 K. The red curve shows the fitting to experimental results (blue points).

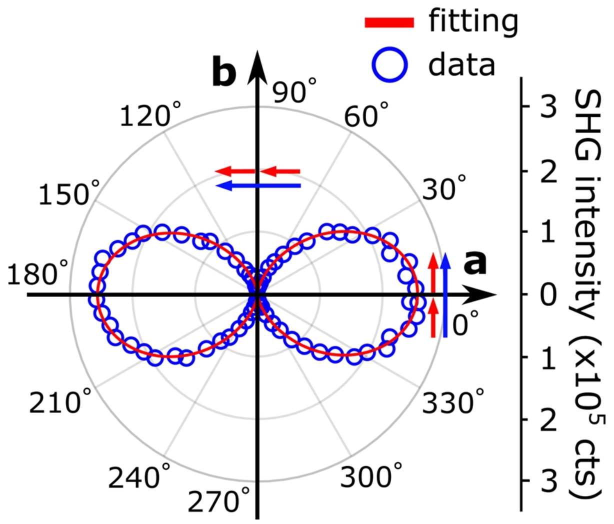

Supplementary Fig. 4. RA-SHG measurements of WTe_2. An azimuthal angle dependent SHG pattern in parallel channel characterizing the reduced crystal symmetry in the *ac*-plane of a WTe_2 nanoflake. The fitting is based on the electric-dipole SHG response under the *m* point group. Red and blue arrows indicate the polarizations of incident and reflected lights at the 0° and 90°, respectively.

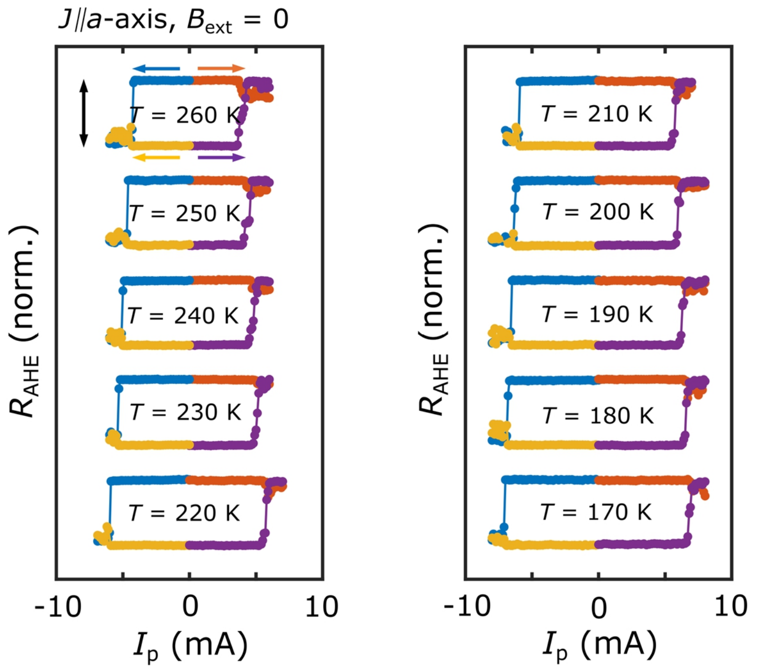

Supplementary Fig. 5. Field-free deterministic magnetic switching of Fe_3GaTe_2. Normalized anomalous Hall resistance of Fe_3GaTe_2 as a function of electrical write current pulse(s) I_p applied along the *a*-axis of WTe_2 recorded at temperatures from 170 K to 260 K. External magnetic field is absent in the measurements. The red and purple (blue and yellow) curves represent I_p swept from zero to the maximum (minimum) value with the magnetic state initialized at $m_z = +1$ and $m_z = -1$, respectively. The black arrow shows the anomalous Hall difference between $m_z = -1$ and $m_z = +1$.

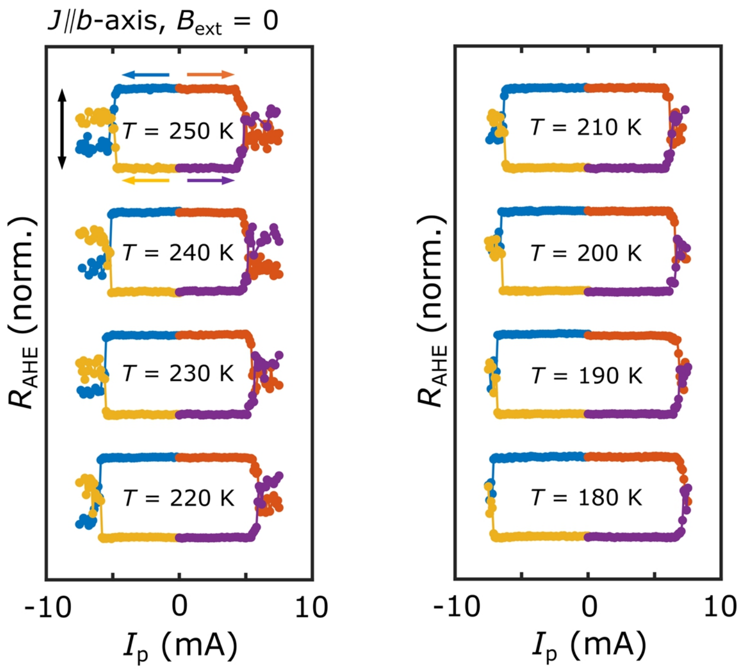

Supplementary Fig. 6. Indeterministic control of Fe_3GaTe_2 magnetization. Normalized anomalous Hall resistance of Fe_3GaTe_2 as a function of electrical write current pulse(s) I_p applied along the *b*-axis of WTe_2 recorded at temperatures from 180 K to 250 K. External magnetic field is absent in the measurements. The red and purple (blue and yellow) curves represent I_p swept from zero to the maximum (minimum) value with the magnetic state initialized at $m_z = +1$ and $m_z = -1$, respectively. The black arrow shows the anomalous Hall difference between $m_z = -1$ and $m_z = +1$.

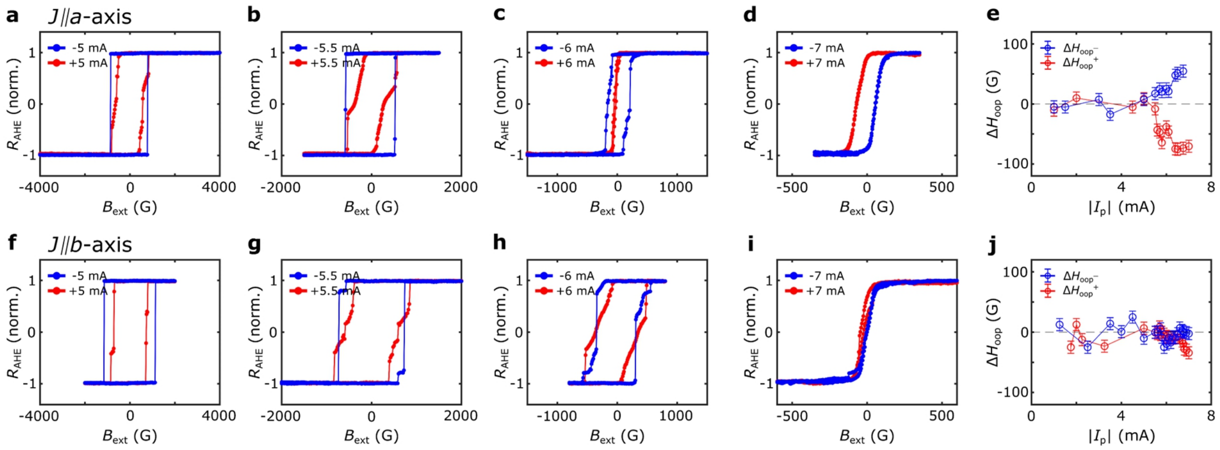

Supplementary Fig. 7. Characterization of out-of-plane effective SOT fields from WTe_2. a-d, Anomalous Hall loops of an Fe_3GaTe_2 sample in response to positive and negative electric current pulses $\left|I_{\mathrm{p}}\right|$ with a magnitude of 5 mA (**a**), 5.5 mA (**b**), 6 mA (**c**), and 7 mA (**d**) applied along the *a*-axis of WTe_2. **e**, Out-of-plane effective SOT field ΔH_{oop} measured as a function of $\left|I_{\mathrm{p}}\right|$ when current pulses applied along the *a*-axis of WTe_2. $\Delta H_{\mathrm{oop}}^{+}$ and $\Delta H_{\mathrm{oop}}^{-}$ denote the shift of anomalous Hall loops of Fe_3GaTe_2 for positive ($I_{\mathrm{p}} > 0$) and negative ($I_{\mathrm{p}} < 0$) current pulses, respectively. **f-i**, Anomalous Hall loops of Fe_3GaTe_2 measured under positive and negative electric current pulses $\left|I_{\mathrm{p}}\right|$ with a magnitude of 5 mA (**f**), 5.5 mA (**g**), 6 mA (**h**), and 7 mA (**i**) applied along the *b*-axis of WTe_2. **j**, Out-of-plane effective SOT field ΔH_{oop} measured as a function of $\left|I_{\mathrm{p}}\right|$ when current pulses are applied along the *b*-axis of WTe_2. All the presented data are recorded at 200 K.

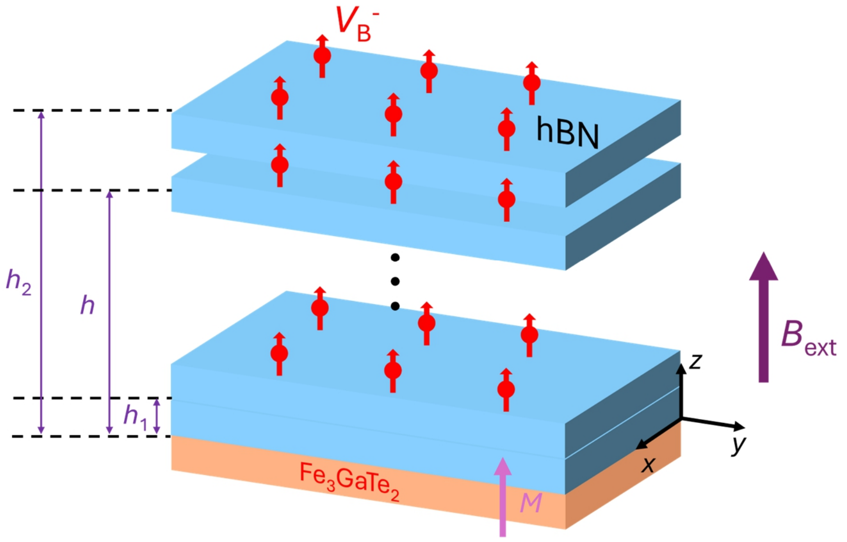

Supplementary Fig. 8. Schematic coordinate system for analyzing Fe_3GaTe_2 magnetization reconstruction. hBN and Fe_3GaTe_2 flakes lie in the *x*-*y* plane and V_B^- spin ensembles are arranged on individual atomic layers of the hBN flake. Thickness of the hBN flake is h_2. Fe_3GaTe_2 magnetization is spontaneously ordered along the *z*-axis direction, which is the same as the V_B^- spin orientation.

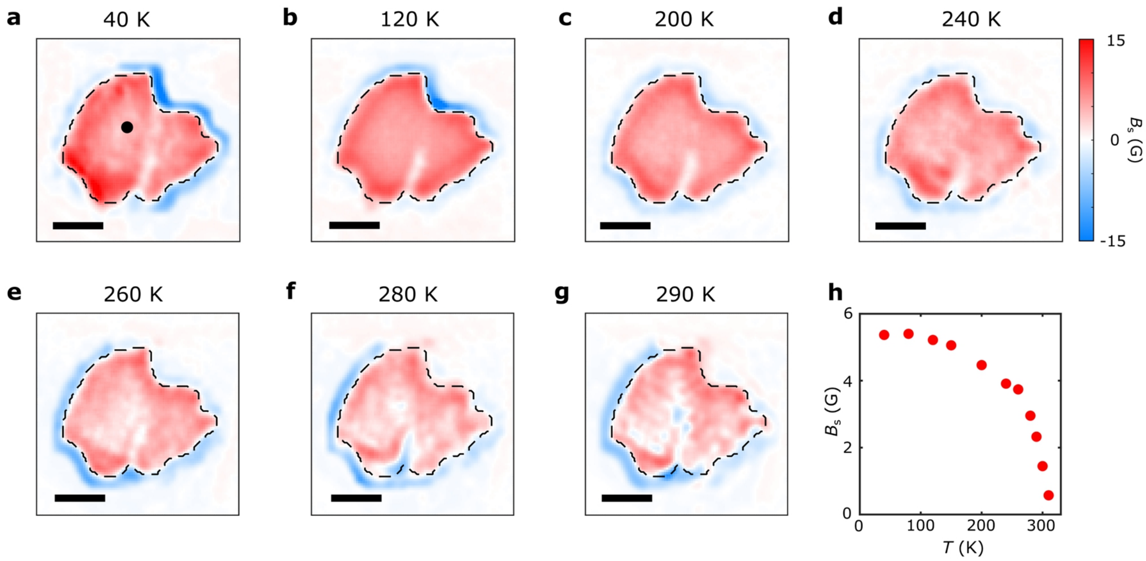

Supplementary Fig. 9. Temperature dependence of magnetic stray fields emanating from Fe_3GaTe_2. a-g, 2D stray field B_s maps of an exfoliated Fe_3GaTe_2 flake (device A) measured with a perpendicular magnetic field B_{ext} = 30 G at 40 K (**a**), 120 K (**b**), 200 K (**c**), 240 K (**d**), 260 K (**e**), 280 (**f**), and 290 K (**g**). The black dashed lines outline the boundary of the Fe_3GaTe_2 flake, and the scale bar is 4 μm. **h**, Temperature dependence of magnetic stray field B_s measured at a local sample site (black point shown in **a**) of the Fe_3GaTe_2 flake.

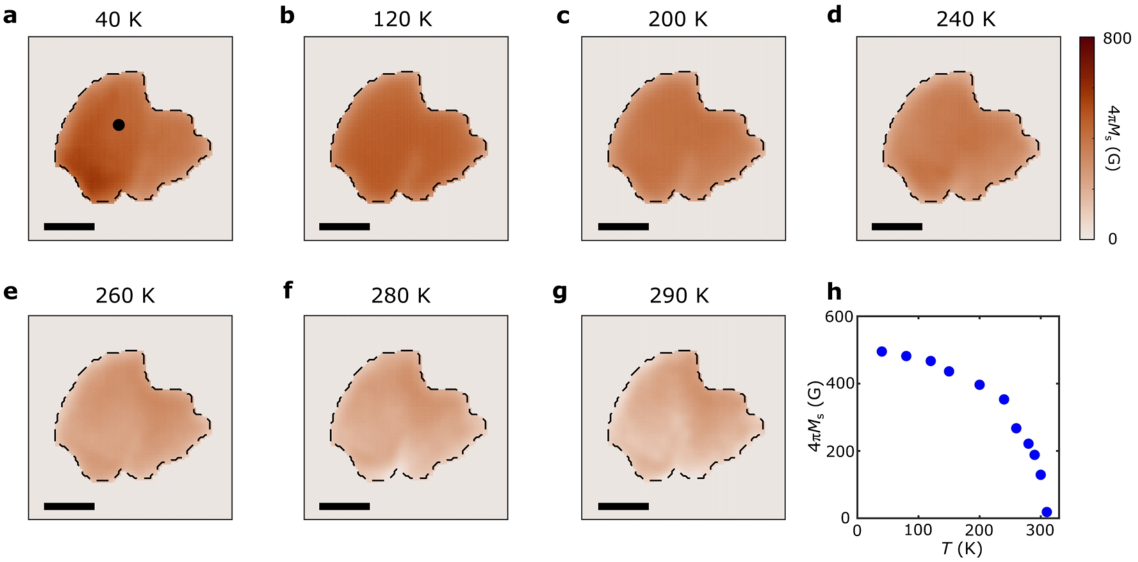

Supplementary Fig. 10. Temperature dependent Fe_3GaTe_2 magnetization maps. a-g, Reconstructed magnetization ($4\pi M_s$) maps of the exfoliated Fe_3GaTe_2 flake (device A) measured with a perpendicular magnetic field B_{ext} = 30 G at 40 K (**a**), 120 K (**b**), 200 K (**c**), 240 K (**d**), 260 K (**e**), 280 (**f**), and 290 K (**g**). The black dashed lines outline the boundary of the Fe_3GaTe_2 flake, and the scale bar is 4 µm. **h**, Temperature dependence of magnetization $4\pi M_s$ measured at a local sample site (black point shown in **a**) of the Fe_3GaTe_2 flake.

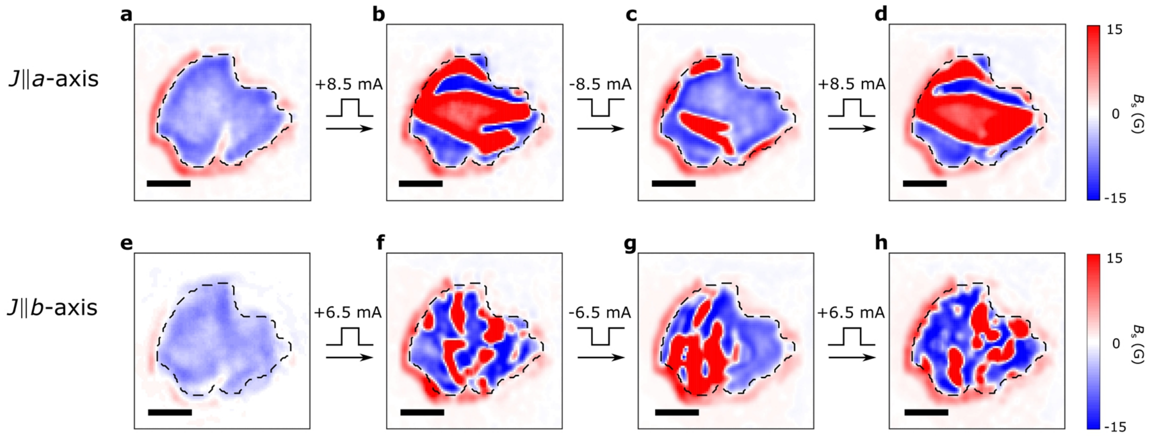

Supplementary Fig. 11. Extended wide-field imaging of deterministic and probabilistic magnetic switching of Fe_3GaTe_2 device A. a-d, Variations of magnetic stray field patterns of the Fe_3GaTe_2 sample (device A) in response to individual electric current pulse applications ($I_p = \pm 8.5$ mA) along the *a*-axis of WTe_2. **e-h**, The Fe_3GaTe_2 sample (device A) shows randomly oriented magnetic domains with approximately equal probabilities of opposite polarities when current pulses $I_p = \pm 6.5$ mA applied along the *b*-axis of WTe_2. The scale bar is 4 μm. The presented quantum sensing measurements are performed at 260 K.

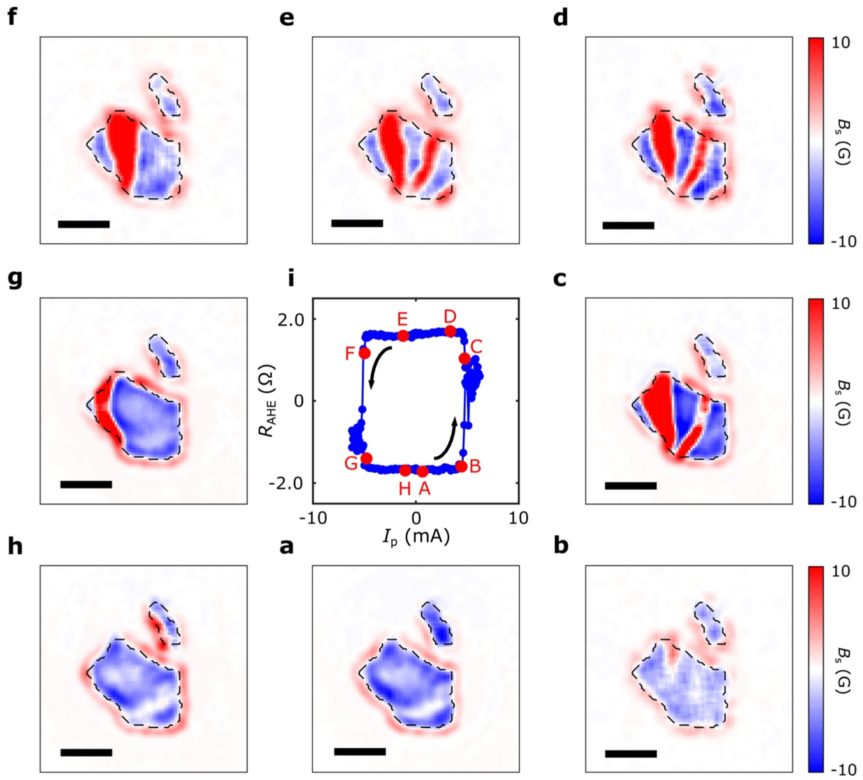

Supplementary Fig. 12. hBN-imaging of field-free deterministic magnetic switching in device B. a-h, Wide-field quantum imaging of microscopic evolution of Fe_3GaTe_2 magnetic domains during the field-free deterministic magnetic switching process. The scale bar is 4 µm. **i**, Anomalous Hall resistance of Fe_3GaTe_2 measured as a function of the current pulse I_p during the field-free deterministic magnetic switching process. The arrows indicate that I_p is swept from zero following the counterclockwise direction around the hysteresis loop and finally returns to the starting point. hBN-based quantum imaging measurements presented in Supplementary Figs. 12a-12h are performed at the corresponding points from "A" to "H" on the magnetic switching loop. The SOT-driven magnetic switching and quantum sensing measurements are performed at 220 K.

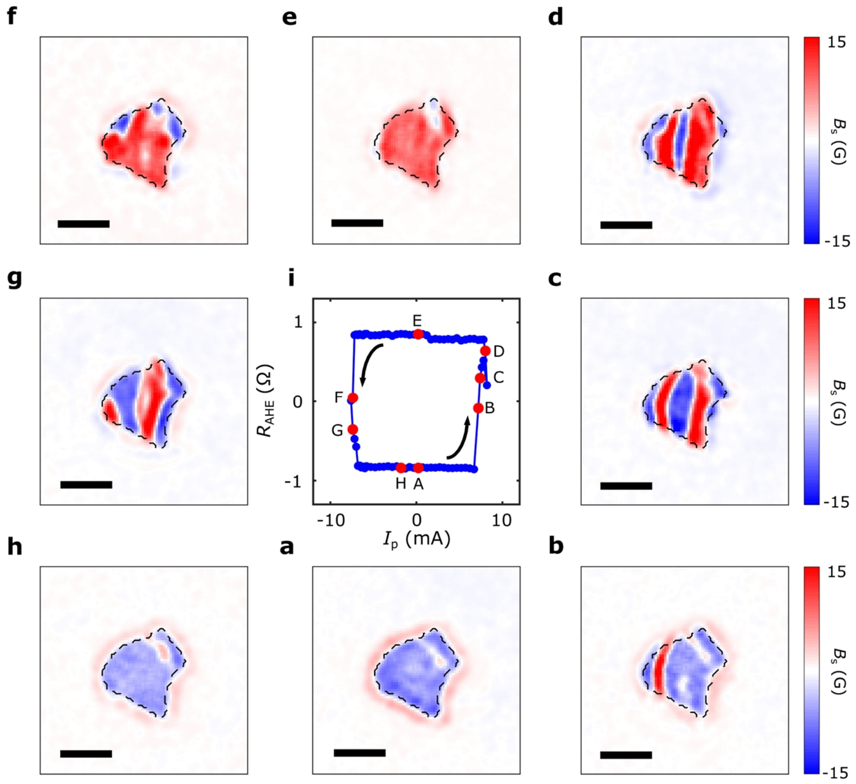

Supplementary Fig. 13. hBN-imaging of field-free deterministic magnetic switching in device C. a-h, Wide-field quantum imaging of microscopic evolution of Fe_3GaTe_2 magnetic domains during the field-free deterministic magnetic switching process. The scale bar is 4 μm. **i**, Anomalous Hall resistance of Fe_3GaTe_2 measured as a function of the current pulse I_p during the field-free deterministic magnetic switching process. The arrows indicate that I_p is swept from zero following the counterclockwise direction around the hysteresis loop and finally returns to the starting point. hBN-based quantum imaging measurements presented in Supplementary Figs. 13a-13h are performed at the corresponding points from "A" to "H" on the magnetic switching loop. The SOT-driven magnetic switching and quantum sensing measurements are performed at 200 K.

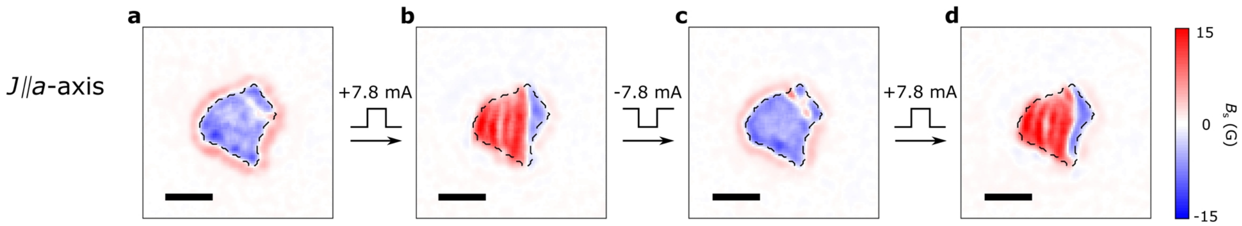

Supplementary Fig. 14. Wide-field imaging of field-free deterministic magnetic switching in device C. a-d, Variations of magnetic stray field patterns of the Fe_3GaTe_2 sample (device C) in response to individual electric current pulse applications ($I_p = \pm 7.8$ mA) along the *a*-axis of WTe_2. The scale bar is 4 μm. The presented quantum sensing measurements are performed at 200 K.

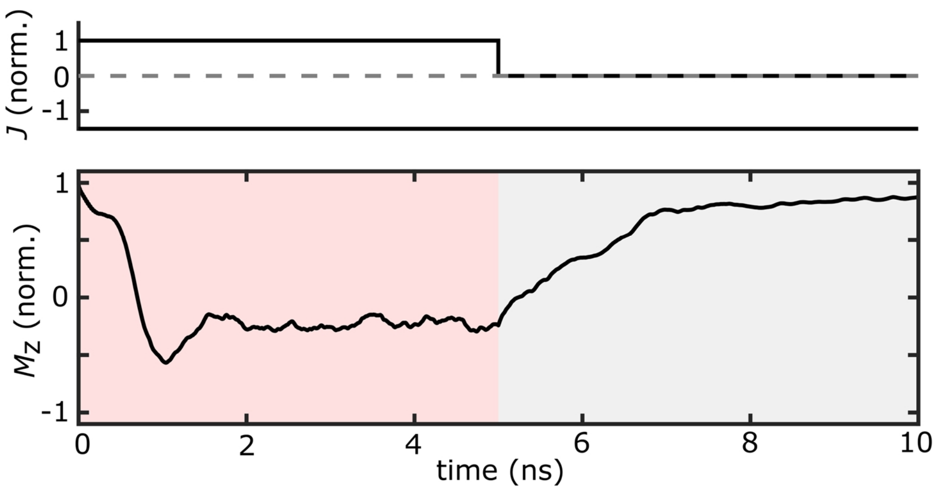

Supplementary Fig. 15. Simulations of stochastic magnetic switching in absence of field-like SOT contribution. Micromagnetic simulations of stochastic magnetic switching of Fe_3GaTe_2 driven by electric current pulses with an amplitude of $J = 2.6 \times 10^{11}$ A/m^2 flowing along the *a*-axis of WTe_2. Normalized current density J (top panel) and magnetization M_z (bottom panel) are shown along the evolution of the spins over time. The field-like SOT efficiency is set to be 0 in presented simulations and other simulation parameters are available in the Method Section.

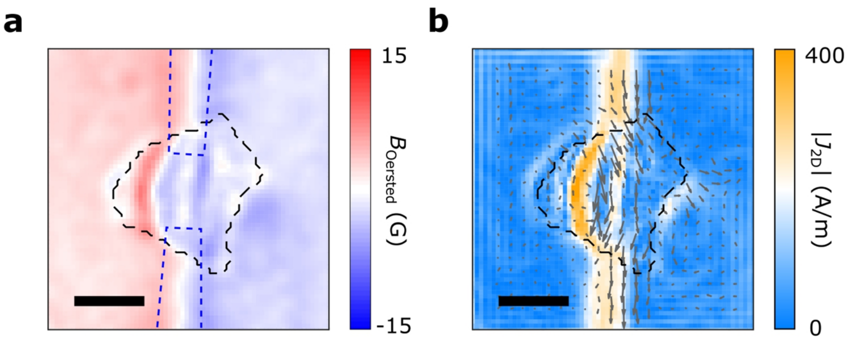

Supplementary Fig. 16. Quantum imaging of microscopic electric current distributions in WTe_2/Fe_3GaTe_2/hBN device C. a, An Oersted field $B_{Oersted}$ map corresponding to an electric current of 3.7 mA applied between two Pt electrodes (highlighted by blue dashed lines) of a WTe_2/Fe_3GaTe_2/hBN device (device C). **b**, Spatially dependent 2D electric current density ($|J_{2D}|$) reconstructed from the Oersted field map. Black dashed lines outline the boundary of the Fe_3GaTe_2 flake. The scale bar is 3 μm.

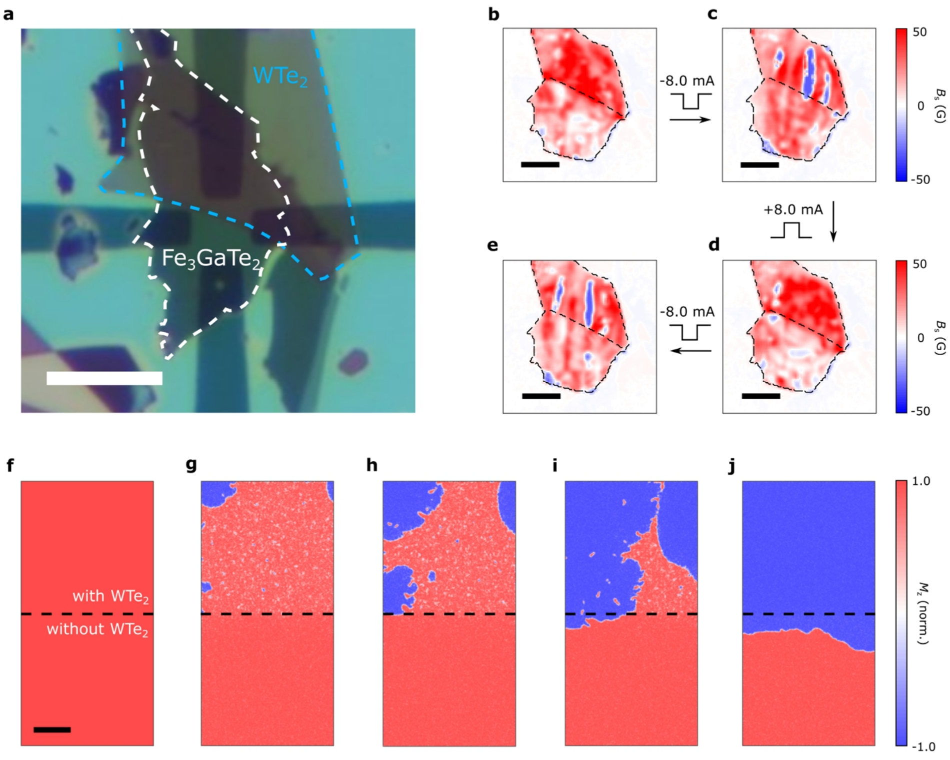

Supplementary Fig. 17. Spatial control of SOT-driven selective magnetic switching in Fe_3GaTe_2. a, Optical microscopy image of a prepared WTe_2/Fe_3GaTe_2/hBN device (device E) with a Fe_3GaTe_2 flake partially covered by a WTe_2 flake. Boundaries of the WTe_2 and Fe_3GaTe_2 flakes are highlighted by blue and white dashed lines, respectively. The scale bar is 10 µm. **b-e**, hBN imaging of local variations of magnetic stray fields of the Fe_3GaTe_2 sample in response to individual electric current pulse applications. The current pulse (I_p) is set to be $\pm$ 8.0 mA applied along the a-axis of WTe_2 in presented measurements. The scale bar is 5 µm. **f-j**, Simulated spatially dependent magnetic switching in a Fe_3GaTe_2 sample partially covered by WTe_2. The Fe_3GaTe_2 sample is present over the entire simulation area while WTe_2 only exists in the bottom half region. Black dashed lines highlight the boundary with/without WTe_2. The amplitude of input electric current pulses density is $J = \pm 1.5 \times 10^{11}$ A/m^2 flowing along the a-axis of WTe_2. Complete magnetization switching is observed in the upper half area of Fe_3GaTe_2 sample covered by WTe_2. Switched magnetic domain extends slightly into the sample region without the WTe_2 coverage due to the local exchange interaction. The ratio η_{FL} between the field-like SOT and damping-like SOT is set to be 0.5 in presented simulations and other simulation parameters are available in the Method Section. Note that variations of η_{FL} between 0.2 and 0.5 provide minor modifications to the simulation results. The scale bar is 0.5 µm.

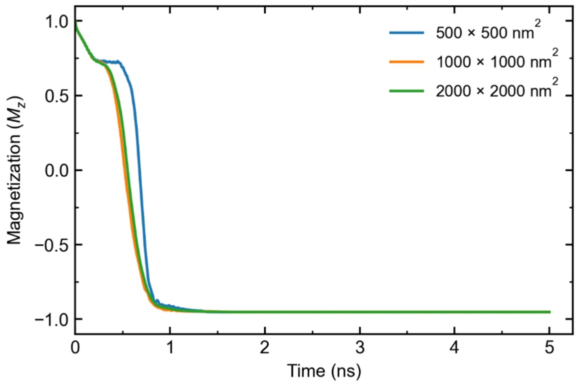

Supplementary Fig. 18. Simulations of size-independent magnetic switching. Simulated time-evolution of the z-component of Fe_3GaTe_2 magnetization (M_z) for varying system dimensions 500 × 500 nm^2, 1000 × 1000 nm^2, and 2000 × 2000 nm^2 under a current density of $J = 2.0 \times 10^{11}$ A/m^2.

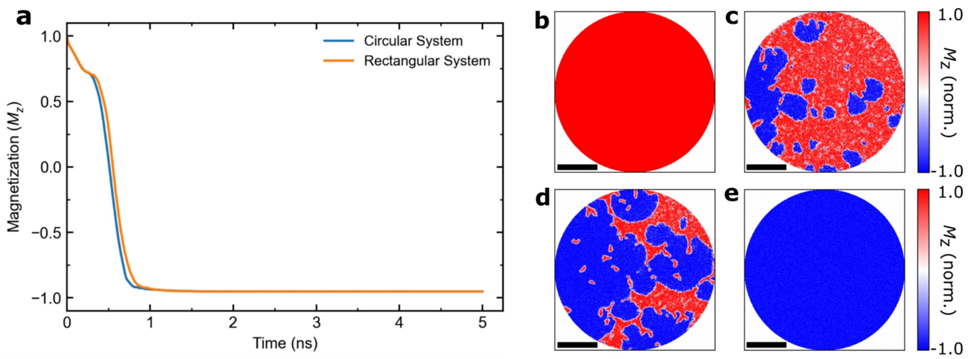

Supplementary Fig. 19. Micromagnetic simulations on circularly shaped Fe_3GaTe_2 sample. **a**, Comparison of the magnetization switching dynamics between a square domain and a circular mesa. Both systems exhibit deterministic reversal at the same critical current density. The nearly overlapping trajectories confirm that the SOT switching mechanism is robust and independent of the specific macroscopic device geometry. **b-e**, Screenshots at different times of the simulated spatially resolved local domain wall nucleation and propagation during the field-free magnetization switching process of a circularly shaped Fe_3GaTe_2 sample. The scale bar is 0.5 μm.
