# Supplementary material for: Imaging of a van der Waals spin-orbit torque system using spin ensembles in hBN
Source: Nat Commun. 2026 Jun 10;17:7350. doi: 10.1038/s41467-026-74178-7 (PMC13402656; doi:10.1038/s41467-026-74178-7)
Supplement: Supplementary file 2 — Description of Additional Supplementary Files [file 41467_2026_74178_MOESM2_ESM.pdf]

Description of Additional Supplementary Files

Supplementary Video 1: Micromagnetic simulations of field-free magnetic switching in Fe_3GaTe_2.
