# Supplementary material for: Imaging of a van der Waals spin-orbit torque system using spin ensembles in hBN
Source: Nat Commun. 2026 Jun 10;17:7350. doi: 10.1038/s41467-026-74178-7 (PMC13402656; doi:10.1038/s41467-026-74178-7)
Supplement: Supplementary file 4 — Transparent Peer Review file [file 41467_2026_74178_MOESM4_ESM.pdf]

nature portfolio

Peer Review File

Imaging of a van der Waals Spin-Orbit Torque System Using Spin Ensembles in hBN

Corresponding Author: Professor Chunhui Du

This manuscript has been previously reviewed at another journal. This document only contains information relating to versions considered at Nature Communications.

This file contains all reviewer reports in order by version, followed by all author rebuttals in order by version.

Version 0:

Reviewer comments:

Reviewer #1

(Remarks to the Author)
In this work, Xi Zhang et al. investigated the SOT switching of $WSe_2/Fe_3GaTe_2/hBN$ heterostructures at room temperature, using both transport measurements and magnetic imaging using boron vacancies inside the hBN layer. The observations are supported by micromagnetic simulations of the switching process and measurements of the current distribution inside the flake during the switching experiments. The paper is nicely written and the results are clear.

I agree with the issues raised by the reviewer 1, field free switching has been performed before using the same materials as in this study $WSe_2/Fe_3GaTe_2/hBN$ (in ref 10 for example) and the imaging of magnetic van der Waals magnets with boron vacancies in hBN has already been demonstrated a few years ago. The authors however added some simulations in Fig. 4 which model the SOT switching and reproduce the data, which seem to indicate an important role of the edges in the switching. The authors conclude that the SOT is necessary to obtain directional switching, by comparing with data obtained by applying stronger current, or applying it along the b axis of WSe_2, thereby triggering a mostly thermal and therefore non directional switching. Although this conclusion seems obvious, the details of both switching processes provided by the authors are interesting. The combined measurements of the magnetization and of the current flow is also a nice experiment.

I think that this work would be better suited to a journal specialized in spintronics or at least condensed matter physics, as the questions which are tackled seem very specific to me and no unknown physical phenomenon or improvement of the capabilities of the imaging methods are presented.

I also have some questions and remarks which can be used to further improve the manuscript:

- In the magnetic field maps, why is there a strong stray field above the whole area of the flake? For an out-of-plane magnetized structure, I would expect field mainly at the edges. The flakes appear rather large, at least 10 µm wide, so even taking into account the limited spatial resolution of the wide field microscope, I would expect a significantly smaller field in the center of the flakes than at the edges (of course except during switching when there is a domain wall). Is it related to the thickness of the sensing layer? To a tilt of the magnetization? I also noticed that in most of the images the contrast around the edges is not the same along the whole contour of the flake, which is not expected since also the out-of-plane component of the magnetic field is measured (for example in Fig. 1g, the blue contrast is stronger on the left of the flake). Is the thickness not uniform?

- Concerning the simulations in Fig 4, the text mentions the role of edges in the switching process, but there is no edge in the depicted simulations! Can the authors explain? As a consequence of the effect of the edges, is the switching efficiency related to the size of the FGaT flake? I understand that only a few samples were prepared, so an experimental answer might be difficult to provide, but does the theory give some insight?

- Can the authors compare the reported SOT efficiency with previously reported values in non van der Waals systems? Is

there actually a gain in the use of van der Waals materials, as the required current densities are similar as those needed in other systems?

Some minor comments :

- Bs is not properly defined.

- When “perpendicular magnetic field” is specified, it is not fully clear if the field is perpendicular to the sample surface or to the VB- axis, in particular in Fig 1. A more precise terminology would be helpful.

Reviewer #2

(Remarks to the Author)
The manuscript reported by Xi Zhang, et al. investigated the hexagonal boron nitride-based quantum imaging of field-free deterministic magnetic switching of room-temperature 2D magnet Fe_3GaTe_2 and spatially resolved electric current distributions in an all-vdW spin-orbit torque (SOT) system. This technology is very important to understand the magnetization switching of the SOT devices. The results presented by the authors look very useful; however, the manuscript did not show the comprehensive results and explanations. Several important things the authors need to be made clear before making the final decision.

1. The results shown in Fig.1d-f are almost same as the results in Ref. 14, I think this is not good thing for this paper. The authors can cite their previous work. In addition, compared the figures between Fig. 1d (middle figure) of Ref. 14 and Fig. 1f of this work, there could be a signal transition, especially 42 G in Ref. 14 and 17 G and 50 G in this work, what is the reason?

2. The field-free SOT magnetization switching of Fe_3GaTe_2 through WTe_2 has been experimentally demonstrated in several literatures, and the authors also presented the hBN-based quantum imaging of the FGT van der Waals ferromagnet in the Ref. 14. Thus the results shown in Fig.1 and Fig.2 are not very important for this work. I think the authors need to give the readers the deeply understanding of the mechanisms of the field-free SOT switching as well as the underline physics through the hBN-based quantum imaging measurements. Some of the data presented in the SI could be the part of the results in the main text.

3. Based on the results of Fig. 2f, we can find that the PMA of Fe_3GaTe_2 is almost damaged when applied ±6.5 mA pulse current (the Coercivity of the Fe_3GaTe_2 is almost zero). In this case, some of the magnetic moment of the Fe_3GaTe_2 could be tilted to some angle, maybe in plane, which also could help magnetization switching by SOT without an external magnetic field. This can also be found in Fig. 3, where we can see that some of regions show the gray or white colors at the initial state as well as the switched state. These results are very useful and interesting; the authors could emphasize this part. In addition, it is not clear for me that why the magnetization moment is starting to switch from the position shown in Fig. 3b, and some of the edge part is not switchable. Based on the image of the device shown in Fig. 1b, the magnetization switching should start from the region of the cross bar. If the authors make the pillar device, what is the situation?

4. For the micromagnetic simulations, the configuration of the magnetization moment and the shape of the device are not fully consistent with the experimental results. The experimental results show that the magnetization moments of some of the regions are not complete pointing down or pointing up, however, the simulation chose the complete down or up magnetization configurations. In addition, the shape of the devices for the simulation should be circle to match with the shape of the pillar devices. In this work, the authors chose the square devices, why? If the shape of the device is circle, what are the results?

5. For the practical application of the SOT device, the shape of the device should be the circle. Thus the valuable results should be the hBN-based quantum images from the circle shape device (pillar devices). In addition, the physical mechanism need to explain deeply based on the experimental and simulation results, which could more valuable for the all-vdW spin-orbit torque device.

6. As mentioned in line 104-106, based on the results of Supplementary Fig 2, "few-layer-thick Fe_3GaTe_2 shows robust magnetic order with the PMA about room temperature", this is not accurate, actually this just confirm the Curie temperature is about room temperature.

Version 1:

Reviewer comments:

Reviewer #1

(Remarks to the Author)
I thank the authors for their detailed answers to all my questions and also to those of the other referee. I my opinion the paper has improved, it is now clearer and more focused on new results. I still think that this work would be better suited for a journal specialized in condensed matter physics, but I have no strong opposition to its publication in Nature Communications.

However, there are two slightly misleading sentences in the current version of the paper which I recommend to amend

before publication:

- lines 102-104: Fe_3GaTe_2 is indeed a room-temperature ferromagnet, but all the experiments presented in this work are realized at lower temperature, I think that this should be reformulated.

- lines 340-342 (last sentence): Sensing using boron vacancies in hBN has indeed the potential to reach van der Waals proximity with the targeted sample, but this is far from achieved here, as the thinnest sensing hBN used is about 30 nm thick. This sentence could actually be removed.

Reviewer #2

(Remarks to the Author)
The authors have addressed all of my comments and suggestions, now I recommend that this manuscript be accepted for publication.

Open Access This Peer Review File is licensed under a Creative Commons Attribution 4.0 International License, which permits use, sharing, adaptation, distribution and reproduction in any medium or format, as long as you give appropriate credit to the original author(s) and the source, provide a link to the Creative Commons license, and indicate if changes were made.
In cases where reviewers are anonymous, credit should be given to 'Anonymous Referee' and the source.
The images or other third party material in this Peer Review File are included in the article's Creative Commons license, unless indicated otherwise in a credit line to the material. If material is not included in the article's Creative Commons license and your intended use is not permitted by statutory regulation or exceeds the permitted use, you will need to obtain permission directly from the copyright holder.
To view a copy of this license, visit https://creativecommons.org/licenses/by/4.0/

Response to reviews − NCOMMS-21-51210A

We thank the reviewers' detailed and constructive assessment of our manuscript. In this revision, we have addressed all the technical questions raised by reviewers, taken their valuable suggestions and revised our manuscript accordingly. A major goal here is to better highlight the key value of our work in revealing new spin-orbit torque (SOT) physics and mechanisms in all-van der Waals (vdW) material systems. Below, we provide a detailed point-by-point response. The reviewers' comments are displayed in *italic* format. Our response is in the black color (Times New Roman) and changes in the main text and Supplementary Information are highlighted in the red color. We hope that the reviewers will find our answers thorough, reasonable, and clear.

Reviewer #1

In this work, Xi Zhang et al. investigated the SOT switching of WSe_2/Fe_3GaTe_2/hBN heterostructures at room temperature, using both transport measurements and magnetic imaging using boron vacancies inside the hBN layer. The observations are supported by micromagnetic simulations of the switching process and measurements of the current distribution inside the flake during the switching experiments. The paper is nicely written and the results are clear.

Response: We thank the reviewer for devoting time and effort to evaluating our manuscript. The reviewer's technical questions are addressed in detail below.

I agree with the issues raised by the reviewer 1, field free switching has been performed before using the same materials as in this study WSe_2/Fe_3GaTe_2/hBN (in ref 10 for example) and the imaging of magnetic van der Waals magnets with boron vacancies in hBN has already been demonstrated a few years ago.

Response: While we agree with the reviewer that field-free magnetic switching in WTe_2/Fe_3GaTe_2 and hBN quantum sensing of vdW magnets have been individually reported before, it is also important to point out that the current work is the first one to experimentally demonstrate a multimodal vdW device platform endowed with dual quantum sensing and electrical SOT functionalities, achieving self-integrated quantum imaging of magnetic switching and electric current flow in all-vdW heterostructures. The advances and development in experimental measurement approaches are clear. We believe that implementation of combined quantum imaging and electrical SOT measurements in a real all-vdW device/material environment will generate interest at the forefront of 2D spintronics, condensed matter physics and quantum sensing research, and thus will be of great interest to the broad readership of *Nature Communications*. This point is also echoed by Reviewer#2 in the previous round review in Nature Materials as quoted below:

> "*If the authors wish to claim that they reveal how the observed magnetic switching evolves, they need to provide more quantitative discussion on the results shown in Fig. 4 as well as establishing general insight based on the experimental results. I think this is not necessary if this manuscript is transferred to Nature Communications.*"

The authors however added some simulations in Fig. 4 which model the SOT switching and reproduce the data, which seem to indicate an important role of the edges in the switching. The

authors conclude that the SOT is necessary to obtain directional switching, by comparing with data obtained by applying stronger current, or applying it along the b axis of WSe_2, thereby triggering a mostly thermal and therefore non directional switching. Although this conclusion seems obvious, the details of both switching processes provided by the authors are interesting. The combined measurements of the magnetization and of the current flow is also a nice experiment.

Response: We thank the reviewer for the detailed and positive assessment of our manuscript.

I think that this work would be better suited to a journal specialized in spintronics or at least condensed matter physics, as the questions which are tackled seem very specific to me and no unknown physical phenomenon or improvement of the capabilities of the imaging methods are presented.

Response: We have carefully considered the reviewer's comments above. Here, we respectfully disagree with the reviewer that "there is no unknown physical phenomenon or improvement of the capabilities of the imaging methods" reported. In fact, the new physics/mechanisms discovered on field-free SOT switching and the novelty of all-vdW-based dual quantum sensing and spintronic platform presented in this work are clear. Below, we would like to share our thoughts to form a two-way communication.

1. **New information**: Taking advantage of hBN-based quantum wide-field microscopy, for the first time, we directly visualize field-free (deterministic) magnetic switching of a room-temperature vdW magnet Fe_3GaTe_2 at the nanoscale. We present SOT-driven local domain wall nucleation and propagation in atomically thin Fe_3GaTe_2. We further resolve spatially dependent electric current distributions in the all-vdW SOT system and report its close correlation with local SOT switching efficiency. This new information, which is otherwise difficult to access by conventional transport measurements, offers valuable insights into material design, testing, and performance evaluation of next-generation vdW spintronic devices.

2. **New SOT physics**: The current study sheds light on new physics/mechanisms on unconventional SOT-driven field-free switching in all-vdW systems. Our simulations show that field-like SOT could provide a low-energy path for local domain wall nucleation and propagation in Fe_3GaTe_2 and plays an important role in inhibiting thermal fluctuation driven stochastic magnetic switching behaviors, which effectively assists in the deterministic magnetization control process. In its absence, magnetic switching evolves into a stochastic process which relies on random thermal fluctuations to overcome the more uniform energy barrier, resulting in far more chaotic switching dynamics. We further image how the observed field-free magnetic switching evolves from deterministic to stochastic nature due to the interplay between spin orientations, anisotropy, crystallographic axis direction and Joule heating in the all-vdW SOT system. To our best knowledge, the new physics discussed above is presented for the first time in the current state of the art.

3. **Multimodal vdW platform**: The advance on measurement approaches reported in the current study is also evident. We present a multimodal vdW device platform endowed with dual quantum sensing and electrical SOT functionalities. For the first time, we achieved *in-situ* quantum imaging and electrical transport measurements in a single vdW stacking device.

We share the optimism that the self-integrated hBN quantum sensing techniques reported here will open new opportunities for exploring nanoscale electromagnetic behaviors in a broad family of miniaturized 2D devices. This also comes back to our point above that experimental demonstration of the cutting-edge imaging techniques together with *in-situ* electrical transport measurements in a real all-vdW device/material platform are of great interest to the fast-advancing quantum sensing, spintronics and condensed matter physics research.

In summary, the experimental approach we report is state-of-the-art, our results are and technically sound, and the immediate scientific impact is clear. We trust that our work meets the high standards of *Nature Communications*, and we hope that the reviewer will share our excitement.

I also have some questions and remarks which can be used to further improve the manuscript:
In the magnetic field maps, why is there a strong stray field above the whole area of the flake? For an out-of-plane magnetized structure, I would expect field mainly at the edges. The flakes appear rather large, at least 10 µm wide, so even taking into account the limited spatial resolution of the wide field microscope, I would expect a significantly smaller field in the center of the flakes than at the edges (of course except during switching when there is a domain wall). Is it related to the thickness of the sensing layer? To a tilt of the magnetization?

Response: We thank the reviewer for this detailed comment. We agree with the reviewer that magnetic stray field should be mainly at edge areas for an out-of-plane magnetized structure. However, due to the finite size effect and spatial resolution of wide-field quantum microscopy, in fact, stray field emanating from the central sample areas is not vanishingly small in micrometer-sized Fe_3GaTe_2 flakes. We have performed micromagnetic simulations to demonstrate this effect as shown in Fig. R1. One can see that the central area of a circular Fe_3GaTe_2 flake also produces a robust magnetic stray field, in qualitative agreement with our experimental results.

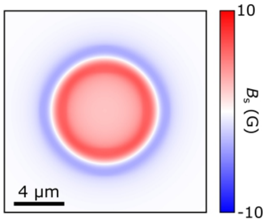

Fig. R1. Micromagnetic simulations of spatial distribution of magnetic stray fields generated by a 2D Fe_3GaTe_2 disk. The vertical distance between the field sensor and sample is 30 nm.

I also noticed that in most of the images the contrast around the edges is not the same along the whole contour of the flake, which is not expected since also the out-of-plane component of the magnetic field is measured (for example in Fig. 1g, the blue contrast is stronger on the left of the flake). Is the thickness not uniform?

Response: We thank the reviewer for this detailed point. The difference of the contrast around samples can be potentially induced by spatially nonuniform magnetization of the Fe_3GaTe_2 flakes. We have also simulated this effect as shown in Fig. R2. It is instructive to note that microscopically inhomogeneous

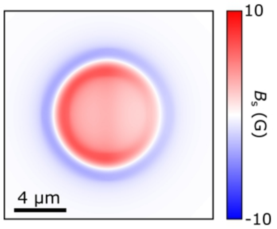

Fig. R2. Micromagnetic simulations of magnetic stray fields generated by a 2D Fe_3GaTe_2 disk with spatial nonuniform magnetization. The vertical distance between the field sensor and sample is 30 nm.

magnetization is commonly observed in atomically thin 2D magnets as reported by previous quantum sensing studies [*Adv. Mater.* **32**, 2003314 (2020), *Science* **364**, 973 (2019), and *Nat. Commun.* **12**, 1989 (2021)] from other leading research groups.

Concerning the simulations in Fig 4, the text mentions the role of edges in the switching process, but there is no edge in the depicted simulations! Can the authors explain?

Response: We thank the reviewer for this detailed point. We believe that the reviewer is referring to the sentence below in the first paragraph on page 6 of the original manuscript.

> "…One can see that the magnetization reversal initiates with nucleation of small local domain drops, which appear predominantly at edges of the sample…"

Here, we clarify that the above sentence was intended to describe our experimental data not micromagnetic simulation results. We apologize for potential confusion caused to readers. In this revision, we have deleted the second half of the sentence as below to make our presentation more accurate.

> "…One can see that the magnetization reversal initiates with nucleation of small local domain drops. The flipped magnetic patches then rapidly expand and merge via domain wall propagation (see Supplementary Video)…"

In the micromagnetic simulations presented, we utilize a 2000 × 2000 nm^2 system size with Periodic Boundary Conditions applied in in-plane directions to model the bulk behavior of the large experimental flake (usually greater than 10 micrometers) while maintaining computational feasibility. To mitigate finite size effects arising from the calculation of the demagnetization field, we include 10 periodic images of the system in the dipolar interaction calculation. Hence, the simulation can be considered as a much larger continuous film, and the boundaries visible in the visualization are merely the limits of the periodic unit cell rather than physical terminations. In this ideal configuration, domain nucleation is driven by stochastic thermal fluctuations, occurring at random Fe_3GaTe_2 sample locations where local energy barriers are momentarily overcome. To account for the stochastic nature, we recalibrate our simulation baseline using 5 random seeds for each configuration, establishing a robust critical switching current.

As a consequence of the effect of the edges, is the switching efficiency related to the size of the FGaT flake? I understand that only a few samples were prepared, so an experimental answer might be difficult to provide, but does the theory give some insight?

Response: We thank the reviewer for raising this insightful point. We did not explicitly discuss the edge effect in the manuscript as it is correlated with other intrinsic/extrinsic material properties/parameters such as sample dimensions/shapes, device quality, local defects, magnetic uniformity, interfacial conditions, local electric current distribution and electrical contact efficiency of Pt electrodes and vdW materials. As one can expect, many of the material properties/parameters mentioned here may vary between different vdW heterostructure devices.

Here, we provide some preliminary insights on the relationship between switching efficiency and flake size from the perspective of micromagnetic theory. In the macroscopic scale relevant to our experiments, where device dimensions are significantly larger than the exchange length and domain wall width, the critical current density is governed primarily by the energy barrier to nucleate a reversed domain. As the edges are spatially decoupled from the bulk in terms of short-range exchange interactions, this nucleation threshold is determined by local edge properties such as demagnetization gradients and defects, rather than the total surface area of the sample. Therefore, for macroscopic flakes, the switching efficiency is expected to remain relatively independent of lateral size.

To further investigate this relationship, we have performed extended simulations across multiple system dimensions: 500 × 500 nm^2, 1000 × 1000 nm^2, and 2000 × 2000 nm^2. We observe that the critical current density required for deterministic switching remained largely consistent across all sample sizes, with successful switching observed in all 5 random seed trials for each case (Fig. R3). These results confirm that finite-size effects are not prominent in the micrometer-scale regime and the switching efficiency is mainly governed by the intrinsic nucleation barrier instead of the total sample area. However, if the device dimensions are reduced to the nanometer scale (e.g., sub-100 nm), finite size effects would become much more significant due to long range dipolar interactions extending across the entire device, modifying the effective anisotropy and making the switching efficiency size dependent. In this revision, we have added Fig. R3 in Supplementary Information Note 12 and the detailed discussion presented above is included in the second paragraph on page 5 as below:

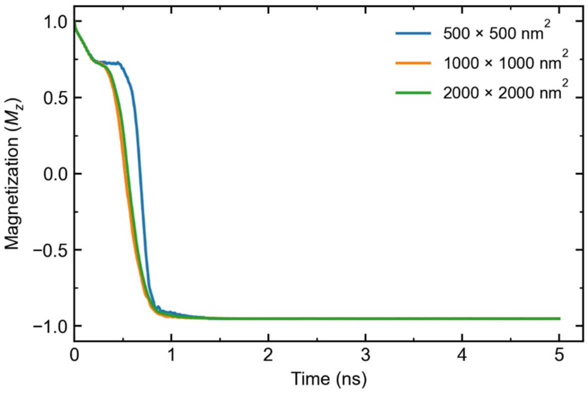

Fig. R3. Simulations of size-independent magnetic switching. Simulated time-evolution of the z-component of magnetization (M_z) for varying system dimensions 500 × 500 nm^2, 1000 × 1000 nm^2, and 2000 × 2000 nm^2 under a current density of J = 2.0 × 10^{11} A/m^2. The switching trajectories are nearly indistinguishable, confirming that finite-size effects are negligible in the micrometer-scale regime and the critical current density is mainly governed by the intrinsic nucleation barrier rather than the total sample area.

> "Here, we briefly comment on the relationship between SOT switching efficiency and flake size from a theoretical perspective. In the macroscopic scale relevant to our experiments, where device dimensions are significantly larger than the exchange length and domain wall width, the critical current density is governed primarily by the energy barrier to nucleate a reversed domain. As the sample edges are spatially decoupled from the bulk in terms of short-range exchange interactions, this nucleation threshold is determined by local edge properties such as demagnetization gradients and defects, rather than the total surface area of the sample. Therefore, for macroscopic flakes, the switching efficiency is expected to remain relatively independent of lateral size. However, if the device dimensions are reduced to the nanometer scale (e.g., sub-100 nm),

finite size effects would become much more significant due to long range dipolar interactions extending across the entire device, modifying the effective anisotropy and making the switching efficiency size dependent.…"

Can the authors compare the reported SOT efficiency with previously reported values in non van der Waals systems? Is there actually a gain in the use of van der Waals materials, as the required current densities are similar as those needed in other systems?

Response: A similar question was also raised in the previous round of review in Nature Materials. Here, we add that the SOT efficiency (when electric current flows along the *a*-axis of WTe_2) is estimated to be ~0.180 in the all-vdW material system in the current work (see Supplementary Information Note 6 for details), which is comparable with that of 5*d* heavy metals such as Pt, Ta and W used in non-vdW systems.

Regarding the gain of vdW materials in spintronics applications, it is instructive to note that the associated advantages not only depend on the nominal SOT efficiency, but also result from some other unrivaled benefits of artificial 2D stacking systems such as the convenient vdW material co-integration strategy, layer dependent magnetic control, stacking tunable lattice interaction, highly engineerable vdW interfacial conditions, readily established vdW proximity and others. We hope that the current work will stimulate more research interest and efforts along this fast-advancing research direction and contribute to the development of next-generation spintronics innovations.

Some minor comments:
Bs is not properly defined.

Response: In our paper, B_s denotes the out-of-plane component of magnetic stray field generated by Fe_3GaTe_2 flake(s). It is defined in the second paragraph on page 3 of the original main text. In this revision, we further define B_s and its direction when it first appears in the figure captions.

When "perpendicular magnetic field" is specified, it is not fully clear if the field is perpendicular to the sample surface or to the VB- axis, in particular in Fig 1. A more precise terminology would be helpful.

Response: We thank the reviewer for pointing out the inaccuracy in our presentation. The "perpendicular magnetic field" refers to magnetic field with the direction perpendicular to the sample plane. We have updated the relevant expressions to make them accurate and consistent throughout the manuscript as below:

"…The measurement temperature is 260 K with an out-of-plane oriented external magnetic field B_{ext} of 30 G applied..."

Reviewer #2:

The manuscript reported by Xi Zhang, et al. investigated the hexagonal boron nitride-based quantum imaging of field-free deterministic magnetic switching of room-temperature 2D magnet

Fe_3GaTe_2 and spatially resolved electric current distributions in an all-vdW spin-orbit torque (SOT) system. This technology is very important to understand the magnetization switching of the SOT devices. The results presented by the authors look very useful; however, the manuscript did not show the comprehensive results and explanations. Several important things the authors need to be made clear before making the final decision.

Response: We thank the reviewer for the detailed evaluation of our work. The reviewer's technical questions are addressed below.

1. The results shown in Fig.1d-f are almost same as the results in Ref. 14, I think this is not good thing for this paper. The authors can cite their previous work. In addition, compared the figures between Fig. 1d (middle figure) of Ref. 14 and Fig. 1f of this work, there could be a signal transition, especially 42 G in Ref. 14 and 17 G and 50 G in this work, what is the reason?

Response: We thank the reviewer for this constructive point. In this revision, the original Fig. 1d is deleted and Fig. 1f is moved to Supplementary Information Note 3. The original Fig. 1e and Fig. 1g are also rearranged accordingly (see our response below for details). We hope that this revision will make our paper concise, coherent, and avoid delivering similar information to the readership/community.

2. The field-free SOT magnetization switching of Fe_3GaTe_2 through WTe_2 has been experimentally demonstrated in several literatures, and the authors also presented the hBN-based quantum imaging of the FGT van der Waals ferromagnet in the Ref. 14. Thus the results shown in Fig.1 and Fig. 2 are not very important for this work.

Response: We thank the reviewer for this constructive point on improving the presentation of our manuscript. The purpose of our original Figs. 1-2 is to demonstrate the multimodal vdW device platform with dual electrical SOT switching and self-integrated quantum sensing capabilities. Considering the reviewer's comments, in this revision, we have combined the original Fig. 1 and Fig. 2 to form a new Fig. 1. Some of the nonessential parts (figures) are moved to Supplementary Information to highlight the interesting elements of the latter sections concerning the new SOT mechanism/physics of field-free Fe_3GaTe_2 switching, which are the key values of this work. We have also revised our manuscript and improved the presentation accordingly based on the updated Fig. 1.

I think the authors need to give the readers the deeply understanding of the mechanisms of the field-free SOT switching as well as the underline physics through the hBN-based quantum imaging measurements. Some of the data presented in the SI could be the part of the results in the main text.

Response: We fully agree with the reviewer that our manuscript should focus on the new physics and insights of field-free SOT switching revealed by hBN quantum imaging measurements. Here, we would like to take this opportunity to recapture the new SOT physics/mechanisms presented in the current work.

1. Taking advantage of hBN-based quantum wide-field microscopy, for the first time, we directly visualize field-free (deterministic) magnetic switching of a room-temperature vdW magnet Fe_3GaTe_2 at the nanoscale. We present SOT-driven local domain wall nucleation and

propagation in atomically thin Fe_3GaTe_2 and show how the observed field-free magnetic switching evolves from deterministic to stochastic nature due to the interplay between spin orientations, magnetic anisotropy and Joule heating.

2. Our micromagnetic simulations show that field-like SOT plays an important role in shaping the energy landscape and inhibiting thermal fluctuation driven stochastic magnetic switching, which effectively assists in the deterministic field-free magnetization control of the Fe_3GaTe_2 sample. Without the field-like SOT contribution, the Fe_3GaTe_2 magnetization reversal process is fundamentally less stable and fails to reach a fully switched state. The field-free magnetic dynamics evolves into a stochastic process which relies on random thermal fluctuations to overcome the more uniform energy barrier, resulting in a far more chaotic multi-domain competition behavior.

3. We further resolve spatially dependent electric current distributions in the all-vdW SOT system and observe its close correlation with local SOT switching efficiency. This new information, which is otherwise difficult to access by conventional transport measurements, offers valuable insights into material design, testing, and performance evaluation of next-generation vdW spintronic devices.

We thank the reviewer again for the constructive point on improving our presentation. In this revision, we have added in-depth discussion of the new SOT physics/mechanisms on field-free SOT switching of the all-vdW material system (from both theoretical and experimental perspectives). These new findings/insights are presented in a coherent way together with our quantum sensing and micromagnetic simulation results.

3. Based on the results of Fig. 2f, we can find that the PMA of Fe_3GaTe_2 is almost damaged when applied ±6.5 mA pulse current (the Coercivity of the Fe_3GaTe_2 is almost zero). In this case, some of the magnetic moment of the Fe_3GaTe_2 could be tilted to some angle, maybe in plane, which also could help magnetization switching by SOT without an external magnetic field. This can also be found in Fig. 3, where we can see that some of regions show the gray or white colors at the initial state as well as the switched state. These results are very useful and interesting; the authors could emphasize this part.

Response: We thank the reviewer for this excellent point. In the high current regime, it is possible that SOT and/or Joule heating effect could drive certain Fe_3GaTe_2 magnetization tilted away from the out-of-plane direction, creating local sample areas with reduced energy barrier that is beneficial for field-free magnetic switching. In this revision, we have explicitly mentioned this point in the first paragraph on page 5 as follows:

> "…Here, we note that Joule heating and/or defects induced local variations of the Fe_3GaTe_2 magnetization could assist in the initial domain wall nucleation processes which typically happen in the high current-density sample areas defined by Pt electrodes[1]…"

In addition, it is not clear for me that why the magnetization moment is starting to switch from the position shown in Fig. 3b, and some of the edge part is not switchable. Based on the image of the device shown in Fig. 1b, the magnetization switching should start from the region of the cross bar.

Response: We thank the reviewer for this detailed point. If we outline the boundaries of Pt electrodes in Fig. 3b, one can see that the incipient domain wall nucleation and magnetic switching of Fe_3GaTe_2 indeed starts from the crossbar area as the reviewer expected as shown in Fig. R4. Whether a specific sample area is switchable depends on many experimental factors such as the local magnetic anisotropy, defect-induced pinning effect, sample size, local electric current flow and others. The fact that some edge areas of Fe_3GaTe_2 are not switchable as pointed out by the reviewer is an outcome of the combination of these factors. In this revision, we have included extended discussion on this point in the first paragraph on page 5 as follows:

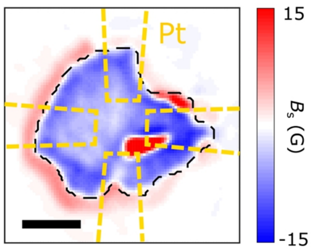

Fig. R4. SOT driven incipient domain wall nucleation and magnetic switching the Fe_3GaTe_2 sample. Yellow dashed lines highlight the boundaries of Pt electrodes. Scale bar is 4 μm.

> "…It is worth mentioning that some edge areas of the Fe_3GaTe_2 flake are not magnetically switchable, which is attributed to the combined effect of sample size, local electric current flow, magnetic anisotropy, pinning effect and others…"

Also, we have added discussion on the relationship between SOT switching efficiency and flake size in the second paragraph on page 5 as follows:

> "Here, we briefly comment on the relationship between SOT switching efficiency and flake size from a theoretical perspective. In the macroscopic scale relevant to our experiments, where device dimensions are significantly larger than the exchange length and domain wall width, the critical current density is governed primarily by the energy barrier to nucleate a reversed domain. As the sample edges are spatially decoupled from the bulk in terms of short-range exchange interactions, this nucleation threshold is determined by local edge properties such as demagnetization gradients and defects, rather than the total surface area of the sample. Therefore, for macroscopic flakes, the switching efficiency is expected to remain relatively independent of lateral size. However, if the device dimensions are reduced to the nanometer scale (e.g., sub-100 nm), finite size effects would become much more significant due to long range dipolar interactions extending across the entire device, modifying the effective anisotropy and making the switching efficiency size dependent…"

If the authors make the pillar device, what is the situation?

Response: If we make micrometer-sized pillar devices, we expect that the general physical picture presented still qualitatively holds except for the fact that electric current flow, sample shape and

size can be controlled in a more precise way in patterned pillar devices. When the lateral dimension of pillar devices shrinks to the nanometer regime (e.g., sub-100 nm), it is instructive to note that macro-spin (single-domain) picture could be potentially involved in describing the magnetic switching processes.

4. For the micromagnetic simulations, the configuration of the magnetization moment and the shape of the device are not fully consistent with the experimental results. The experimental results show that the magnetization moments of some of the regions are not complete pointing down or pointing up, however, the simulation chose the complete down or up magnetization configurations.

Response: In our experiments, the Fe_3GaTe_2 magnetization is fully up or down in the initial state (before any electric current pulse applications). Thus, the configuration of Fe_3GaTe_2 magnetic moment in our simulations qualitatively agrees with the experimental results. It is worth mentioning that variations of local stray field strength shown in original Fig. 3 result from variation of the magnitude, not necessarily the direction, of the out-of-plane Fe_3GaTe_2 magnetization.

Considering some other extrinsic/intrinsic material properties and experimental factors such as vdW interfacial condition, magnetic inhomogeneities, defects, and variations of local electric current flow, we understand that it is difficult (probably impossible) to reproduce the exactly same experimental conditions in our micromagnetic simulations. Given the mutual agreement between our experimental data and simulation results, our model has qualitatively recaptured the major physics and mechanisms of field-free switching in the all-vdW SOT system.

In addition, the shape of the devices for the simulation should be circle to match with the shape of the pillar devices. In this work, the authors chose the square devices, why? If the shape of the device is circle, what are the results?

Response: Selection of a square geometry for the primary simulations is driven by the numerical characteristics of the Finite Difference Method (FDM) employed in our modeling. Since FDM utilizes a rectangular discretization grid, simulating curved geometries might introduce artificial "staircase" roughness at the sample boundaries. These discretization artifacts act as spurious nucleation or pinning sites that do not reflect the physical reality of our exfoliated flakes. By utilizing a square domain with Periodic Boundary Conditions, we effectively model the intrinsic switching limit of the material system without incorporating arbitrary extrinsic factors such as edge roughness or defects.

To directly address the reviewer's request and validate our geometric assumptions, we have conducted extended micromagnetic simulations on a circularly shaped sample. To mitigate the grid discretization artifacts mentioned above, here we apply an advanced edge smoothing algorithm, which utilizes sub-grid averaging to approximate the circular boundary. The simulation results from these circular simulations are qualitatively identical to those of the square samples: we observed deterministic switching in all 5 trials as shown in Fig. R5. Thus, we are convinced that our micromagnetic simulation model (results) are sound and valid, which rationalizes well the experimental results. In this revision, Fig. R5 and the relevant discussion have been included in Supplementary Information Note 12.

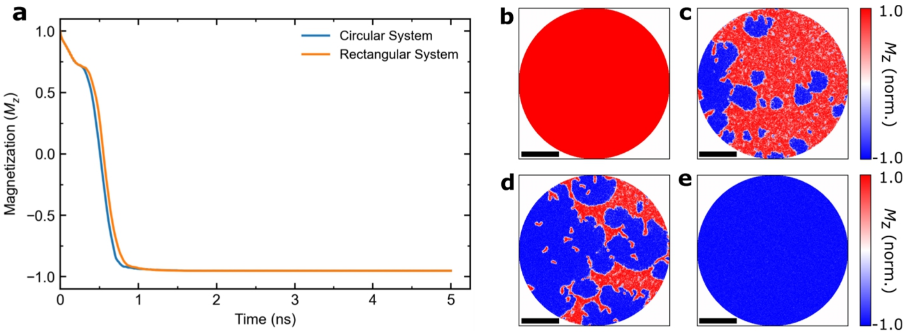

Fig. R5. Micromagnetic simulations on circularly shaped Fe_3GaTe_2 sample. a, Comparison of the magnetization switching dynamics between a square domain (with Periodic Boundary Conditions) and a circular mesa (a circular domain with open boundaries and edge smoothing). Both systems exhibit deterministic reversal at the same critical current density. The nearly overlapping trajectories confirm that the SOT switching mechanism is robust and independent of the specific macroscopic device geometry. **b-e**, Screenshots at different times of simulated spatially resolved local domain wall nucleation and propagation during the field-free magnetization switching process of a circularly shaped Fe_3GaTe_2 sample. The scale bar is 0.5 µm.

5. For the practical application of the SOT device, the shape of the device should be the circle. Thus the valuable results should be the hBN-based quantum images from the circle shape device (pillar devices).

Response: We understand that circularly shaped devices might be beneficial for practical applications. However, technically speaking, the shape of Fe_3GaTe_2 and WTe_2 flakes cannot be precisely controlled in the exfoliation processes of 2D vdW crystals. In the current study, we have tried our best to search for atomically thin Fe_3GaTe_2 and WTe_2 flakes with relatively regular lateral shapes for device fabrication. We also would like to point out that the current work mainly focuses on hBN quantum sensing of field-free magnetization control in all-vdW material systems instead of immediate practical applications. We hope that our work will provide valuable insights into developing cutting-edge vdW spintronic devices, contributing to implementation of next-generation SOT technologies.

In addition, the physical mechanism need to explain deeply based on the experimental and simulation results, which could more valuable for the all-vdW spin-orbit torque device.

Response: We thank the reviewer again for this constructive point on improving our presentation. This also comes back to the reviewer's point 2 above. As detailed in our response above, in this revision, we have added in-depth discussion of the new SOT physics/mechanisms of field-free Fe_3GaTe_2 magnetic switching in the all-vdW system and revised the manuscript accordingly. In particular, we have performed extended micromagnetic simulations and highlight the valuable insights obtained from mutually supported hBN quantum imaging and micromagnetic simulation results, the most interesting part of this manuscript that stands out from previous studies.

We try to avoid repeating our response here. Please find the details in our response to the reviewer's points above.

6. As mentioned in line 104-106, based on the results of Supplementary Fig 2, "few-layer-thick Fe_3GaTe_2 shows robust magnetic order with the PMA about room temperature", this is not accurate, actually this just confirm the Curie temperature is about room temperature.

Response: We thank the reviewer for this detailed point. In this revision, we have revised the sentence accordingly to make it more accurate as follows:

> "…Few-layer-thick Fe_3GaTe_2 shows robust magnetic order above the room temperature (see Supplementary Information Note 2 for details), serving as an excellent material candidate to develop functional vdW spintronic devices for practical applications[10,25,26]…."

In summary, we thank the reviewer again for devoting time and efforts on evaluation our work. We have revised the manuscript based on the detailed and constructive comments. We believe that our study will generate interest at the forefront of quantum sensing, 2D spintronics and condensed matter physics research, and thus will be of great interest to the broad readership of *Nature Communications*.

Response to reviews – NCOMMS-25-78682A

We thank the reviewers' positive assessment and recommendation of our manuscript. In this revision, we have addressed the remaining comments from reviewers and revised our manuscript accordingly. Below, we provide a point-by-point response. The reviewers' comments are displayed in *italic* format. Our response is in the black color (Times New Roman) and changes in the main text and Supplementary Information are highlighted in the red color.

Reviewer #1

I thank the authors for their detailed answers to all my questions and also to those of the other referee. I my opinion the paper has improved, it is now clearer and more focused on new results. I still think that this work would be better suited for a journal specialized in condensed matter physics, but I have no strong opposition to its publication in Nature Communications.

Response: We thank the reviewer' s positive recommendation of our manuscript.

However, there are two slightly misleading sentences in the current version of the paper which I recommend to amend before publication:

- lines 102-104: Fe_3GaTe_2 is indeed a room-temperature ferromagnet, but all the experiments presented in this work are realized at lower temperature, I think that this should be reformulated.

Response: Based on the reviewer's comment, we have deleted the words "for practical applications" and reformulated the language as follows:

> "…Few-layer-thick Fe_3GaTe_2 shows robust magnetic order above the room temperature (see Supplementary Information Note 2 for details), serving as an excellent material candidate to develop functional vdW spintronic logic devices[10,23,24]."

- lines 340-342 (last sentence): Sensing using boron vacancies in hBN has indeed the potential to reach van der Waals proximity with the targeted sample, but this is far from achieved here, as the thinnest sensing hBN used is about 30 nm thick. This sentence could actually be removed.

Response: We have removed this the referred sentence in this final revision.

Reviewer #2

The authors have addressed all of my comments and suggestions, now I recommend that this manuscript be accepted for publication.

Response: We thank the reviewer again for the recommendation of our work.
